# The intellectual disability protein RAB39B selectively regulates GluA2 trafficking to determine synaptic AMPAR composition

**DOI:** 10.1038/ncomms7504

**Published:** 2015-03-18

**Authors:** Maria Lidia Mignogna, Maila Giannandrea, Antonia Gurgone, Francesca Fanelli, Francesco Raimondi, Lisa Mapelli, Silvia Bassani, Huaqiang Fang, Eelco Van Anken, Massimo Alessio, Maria Passafaro, Silvia Gatti, José A. Esteban, Richard Huganir, Patrizia D’Adamo

**Affiliations:** 1 Dulbecco Telethon Institute at IRCCS San Raffaele Scientific Institute, Division of Neuroscience, 20132 Milan, Italy; 2 F. Hoffmann-La Roche AG, pRED Pharma Research & Early Development, DTA Neuroscience, CH4070 Basel, Switzerland; 3 Vita-Salute San Raffaele University, 20132 Milan, Italy; 4 Department of Life Sciences, University of Modena and Reggio Emilia, 41125 Modena, Italy; 5 CNR Institute of Neuroscience, Department of BIOMETRA, University of Milan, 20129 Milan, Italy; 6 Department of Neuroscience and Howard Hughes Medical Institute, Johns Hopkins University School of Medicine, Baltimore, Maryland 21205, USA; 7 IRCCS San Raffaele Scientific Institute, Division of Genetics and Cell Biology, 20132 Milan, Italy; 8 Centro de Biología Molecular Severo Ochoa, Consejo Superior de Investigaciones Científicas/Universidad Autónoma de Madrid, Madrid 28049, Spain

## Abstract

RAB39B is a member of the RAB family of small GTPases that controls intracellular vesicular trafficking in a compartment-specific manner. Mutations in the *RAB39B* gene cause intellectual disability comorbid with autism spectrum disorder and epilepsy, but the impact of RAB39B loss of function on synaptic activity is largely unexplained. Here we show that protein interacting with C-kinase 1 (PICK1) is a downstream effector of GTP-bound RAB39B and that RAB39B-PICK1 controls trafficking from the endoplasmic reticulum to the Golgi and, hence, surface expression of GluA2, a subunit of alpha-amino-3-hydroxy-5-methyl-4-isoxazole propionic acid receptors (AMPARs). The role of AMPARs in synaptic transmission varies depending on the combination of subunits (GluA1, GluA2 and GluA3) they incorporate. RAB39B downregulation in mouse hippocampal neurons skews AMPAR composition towards non GluA2-containing Ca^2+^-permeable forms and thereby alters synaptic activity, specifically in hippocampal neurons. We posit that the resulting alteration in synaptic function underlies cognitive dysfunction in RAB39B-related disorders.

## Abstract (web summary)

Mutations in the *RAB39B* gene, which encodes a protein involved in vesicular trafficking, are associated with intellectual disability, but the impact of RAB39B loss of function on synaptic activity is not known. Here the authors show that RAB39B interacts with PICK1, and that this interaction is critical for the translocation of AMPA receptor subunits into the Golgi.

## Graphical abstract

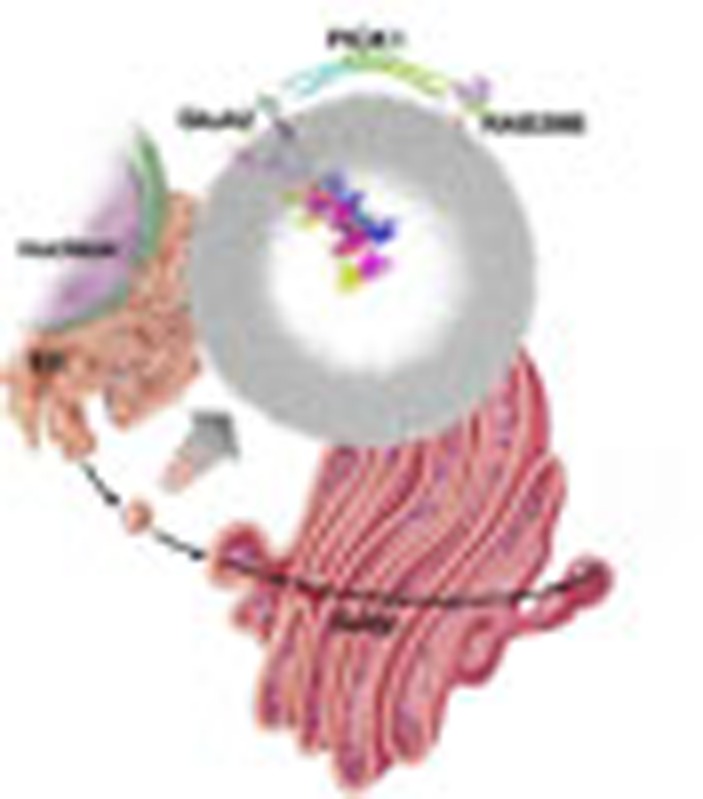

The human *RAB39B* gene, which maps to the distal Xq28 locus, encodes RAB39B, a member of the RAB GTPases, small monomeric GTPases belonging to the RAS-like GTPase superfamily, that have a key role in the regulation of intracellular vesicular trafficking^1^. Indeed, RAB GTPases are physically associated with specific organelles, through specific effector protein interaction, and act as a network to regulate both spatially and temporally the transport of specific vesicles switching from the active GTP-bound and the inactive GDP-bound state^2^,^3^. RAB and RAB-associated proteins have been shown to play an important role in a number of rare monogenic as well as multifactorial diseases^4^,^5^ characterized by cognitive deficit. In particular, previous work on X-linked intellectual disability (XLID) has identified mutations in the *GDI1* gene that cause loss of function of the RAB-interacting protein αGDI^6^,^7^. RAB GTPases and interacting proteins are abundantly expressed in the central nervous system across species, and *Gdi1* mouse models have been relevant to demonstrate the causal link of αGDI loss of function with deficits in glutamate release and cognitive impairment^8^,^9^. Our group previously demonstrated that loss-of-function mutations in human *RAB39B* gene are associated with XLID comorbid with autism spectrum disorder and seizures^10^ and also comorbid with early onset of Parkinson’s disease^11^. Moreover, it was reported that also a 0.5-Mb tandem copy-number gain at distal Xq28 including *RAB39B* was linked to XLID^12^. We defined that RAB39B is a neuronal-specific protein localized at the Golgi compartment, but its functional role is unknown^10^. The demonstrated link with a human genetic disease urged us to define the functional role of RAB39B in neurons and the potential link to cognitive function.

In the present study, we define that RAB39B interacts with protein interacting with C-kinase 1 (PICK1), which became an attractive target for further studies. Previous studies defined the functional role of PICK1 in synaptic alpha-amino-3-hydroxy-5-methyl-4-isoxazole propionic acid receptor (AMPAR) surface expression, trafficking and post-synaptic targeting^13^,^14^,^15^,^16^,^17^,^18^, but the mechanism of AMPAR biogenesis, assembly and trafficking is poorly understood. In hippocampal CA1 pyramidal neurons, AMPARs are hetero-tetramers formed in the endoplasmic reticulum (ER)^19^,^20^,^21^ mostly composed of GluA1/GluA2 or GluA2/GluA3 hetero-dimers^22^. Export of AMPARs from the ER requires the interaction of the C-terminal domain of AMPAR subunits with other proteins. In fact, synapse-associated protein 97 (SAP97) interacts with the immature GluA1 C-terminal domain^23^ early in the secretory pathway^24^, while GluA2 C-terminal domain interacts with PICK1, which is necessary for GluA2 exit from the ER. Thus, PICK1 selectively binds GluA2/GluA3 heterodimers for exit from the ER to Golgi compartment^25^.

Given the RAB39B–PICK1 interaction and the Golgi localization of RAB39B, here we hypothesize that RAB39B might be a key molecule fundamental to regulate a specific secretory trafficking step of GluA2 AMPAR subunit. Supporting this idea, *Rab39b*-silenced mouse hippocampal neurons show increased GluA2 immature form suggesting ER retention. This result provokes, as a final step, a reduction in the amount of GluA2 AMPAR subunits at the post-synaptic membrane, leading to the formation of GluA2-lacking, Ca^2+^-permeable AMPARs, often associated with immature synapses and cognitive impairment^26^.

We report here that RAB39B is the key molecule regulating the translocation of GluA2/GluA3 heterotetramers into the Golgi. Indeed, in the absence of RAB39B, AMPARs arrangement at the post-synaptic site is misregulated, which provides an explanation for the involvement of *RAB39B* mutations in the aetiology of ID and autism spectrum disorder.

## Results

### GTP-bound RAB39B interacts with PICK1

**Figure 1:**
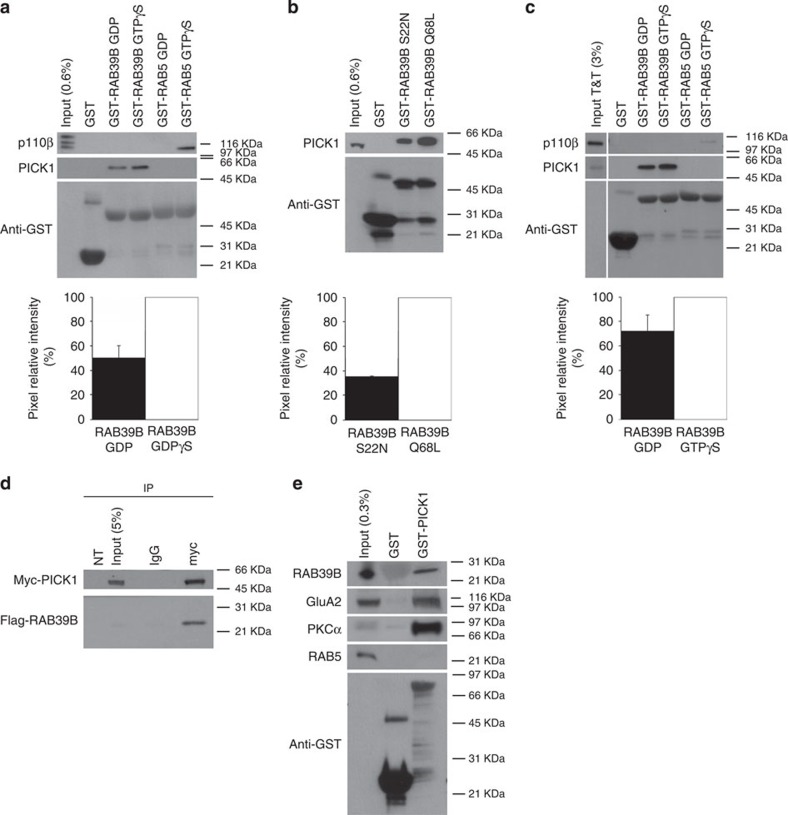
RAB39B interacts with PICK1. (**a**–**c**, upper panel) Representative western blots (*n*=3 experimental replicates) of GST-RAB39B GDP/GTPγS and GST-RAB39B S22N/Q68L pull down. (**a**,**b**) Input: mouse brain lysate. (**c**) Input T&T: PICK1 *in vitro* transcribed and translated. (**a**–**c**, lower panel) Histograms show the percentage±s.e.m. of PICK1 binding to GST-RAB39B GDP versus GST-RAB39B GTPγS. (**d**) Representative western blot (*n*=3 experimental replicates) of co-immunoprecipitation of myc-PICK1 and flag-RAB39B. Input: HEK293T cells; NT: non-transfected cells. (**e**) Representative western blots (*n*=3 experimental replicates) of GST-PICK1 pull down. Input: mouse brain lysate.

To understand the causal link between XLID and gene defects associated with RAB39B, we set out to map the role of RAB39B in vesicular trafficking. As a first step, we searched for downstream effectors of RAB39B by use of a yeast two-hybrid screening (Supplementary Fig. 1a–c). With the human full-length RAB39B protein as bait, by screening a human fetal brain cDNA library we identified a protein originally described as protein interacting with C-kinase 1 (PICK1) as the strongest interacting molecule. In agreement with our results from the two-hybrid screen, recombinant GST-RAB39B locked in the active state with the non-hydrolysable GTP analogue GTPγS and the dominant active GST-RAB39B Q68L efficiently pulled down PICK1, as compared with GDP-bound or dominant-negative GST-RAB39B S22N, using whole-brain lysate of murine origin (Fig. 1a,b). Instead, recombinant GST-RAB39A, sharing 76% identity at protein level with RAB39B, did not pull down PICK1 (Supplementary Fig. 1d).

GST-RAB39B GTPγS likewise pulled down PICK1, obtained by *in vitro* transcription and translation, which indicated that PICK1 interacts directly with RAB39B, and mostly in its GTP-bound state (Fig. 1c). Furthermore, myc-tagged PICK1 co-immunoprecipitates with flag-tagged RAB39B from lysates of HEK293T cells in which the two constructs were co-expressed (Fig. 1d). Finally, we performed the reverse pull down using GST-PICK1 and found that it pulled down RAB39B from the mouse brain lysate, as well as previously identified interacting proteins PKCα^27^,^28^ and the GluA2 AMPAR subunit^29^ (Fig. 1e).

### RAB39B effector-binding region interacts with PICK1 PDZ domain

**Figure 2:**
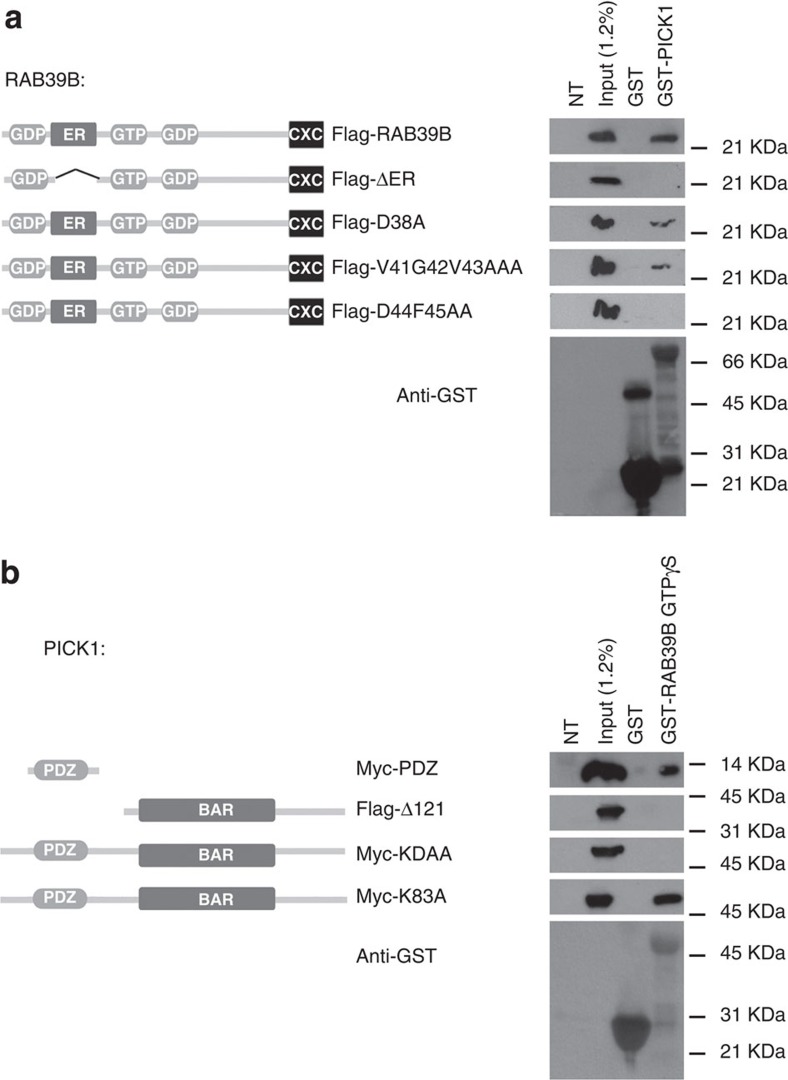
RAB39B–PICK1 interacting domains. Representative western blots (*n*=3 experimental replicates) of GST-pull down using (**a**) GST-PICK1 and (**b**) GST-RAB39B GTPγS. Input: COS7 cells transfected with differently tagged RAB39B and PICK1 constructs. NT: non-transfected cells.

To define how the two proteins interact at the molecular level, COS7 cells were transfected with RAB39B or PICK1 mutant constructs and interactions were tested again by pull down assays. Since GST-PICK1 did not pull down RAB39B construct lacking a stretch (flag-ΔER, aa 35–49) corresponding to the canonical effector-binding region as present in all RAB proteins^30^, we concluded that the association between RAB39B and PICK1 is a conventional RAB-effector protein interaction (Fig. 2a). To better define the residues involved in the PICK1-RAB39B effector-binding region, RAB39B constructs carrying different point mutations were used (flag-D38A, flag-V41G42V43AAA or flag-D44F45AA). Such RAB39B constructs were generated based on the predicted structural complex between RAB39B and PICK1 PDZ (see below). Consistently with structure predictions, GST-PICK1 did not pull down RAB39B construct carrying D44 and F45 mutated to alanines (aa D44F45AA), indicating that such amino-acid pair in the N-terminal half of the β2-strand of RAB39B play a central role in the association between RAB39B and PICK1 (Fig. 2a). PICK1 is a PDZ- and BAR-domain-containing protein. GST-RAB39B GTPγS pulled down recombinant myc-tagged PDZ domain (myc-PDZ; aa 23–99), but not flag-tagged recombinant PICK1 lacking the PDZ domain (flag- Δ 121, aa 1–121; Fig. 2b). Thus, the interface between the two interacting proteins involves amino acids D44 and F45 of the ER domain of RAB39B and, respectively, the PDZ domain of PICK1.

The PDZ domain of PICK1 has been shown before to interact with a variety of proteins^31^,^32^, and point mutations within the PDZ domain of lysine 83 (K83) or of both lysine 27 (K27) and aspartate 28 (D28) with alanines abolished protein binding^29^,^31^,^33^,^34^. Since GST-RAB39B GTPγS pulled down full-length myc-PICK with the K83A substitution (myc-K83A), but not with the K27D28AA substitutions (myc-KDAA), we concluded that K27 and D28 in the PDZ domain is key for the interaction of PICK1 with RAB39B (Fig. 2b).

**Figure 3:**
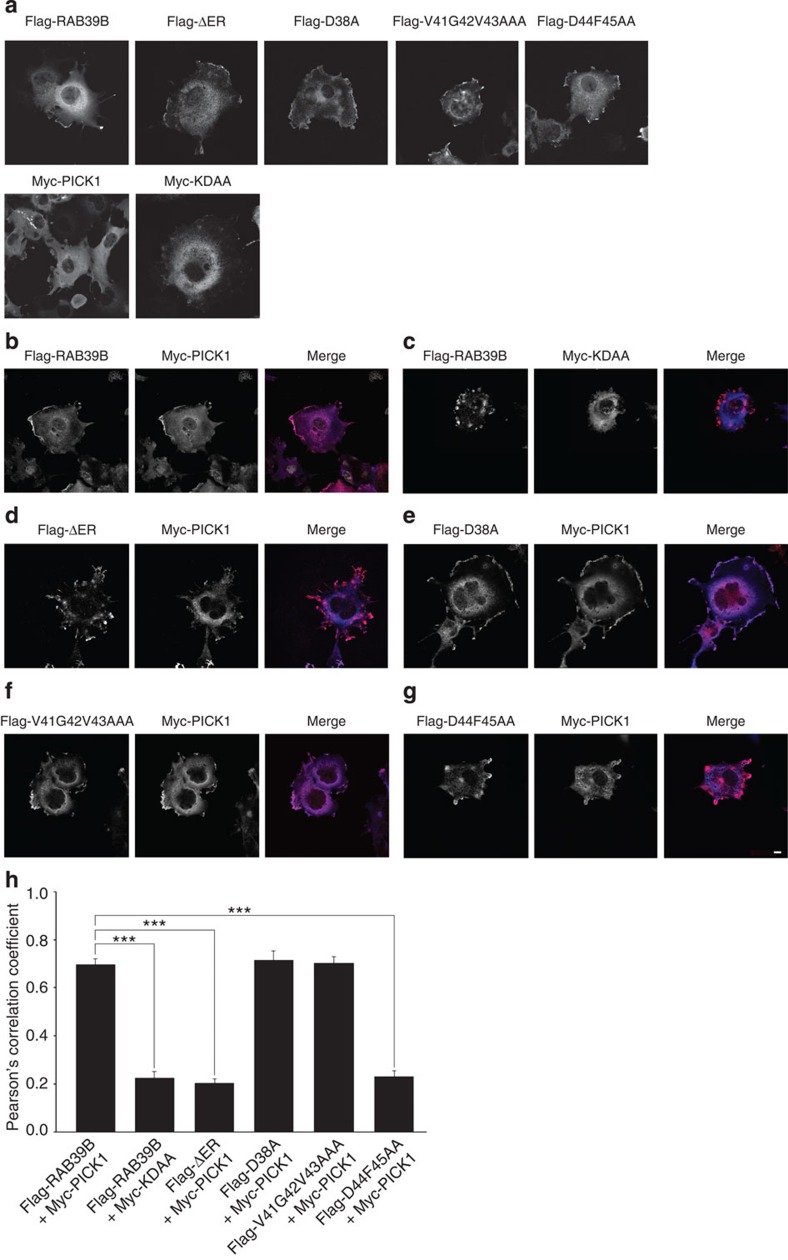
Validation of RAB39B–PICK1 interaction in COS7 cells. Representative immunofluorescence images of COS7 cells (**a**) single or (**b**–**g**) double transfected with different RAB39B (flag-RAB39B, red) and/or PICK1 (myc-PICK1, blue) constructs. Scale bar, 10 μm. (**h**) Histogram shows the Pearson’s correlation coefficients (PCC; means±s.e.m.) for each co-transfection. Significant statistical differences were found comparing flag-RAB39B/myc-PICK1 PCC (*n*=14 cells; 3 experimental replicates; PCC=0.70) to flag-RAB39B/myc-KDAA PCC (*n*=23 cells; three experimental replicates; PCC=0.22, *P* adj.=1.7E−08), to flag-ΔER/myc-PICK1 PCC (*n*=27 cells; three experimental replicates; PCC=0.2, *P* adj.=1.7E−08), and to flag-D44F45AA/myc-PICK1 PCC (*n*=14 cells; 3 experimental replicates; PCC=0.23, *P* adj.=1.7E−08). Benjamini–Hochberg procedure used to test statistical significance. ***P<0.001.

In agreement with our *in vitro* binding assays, we co-expressed flag-RAB39B and myc-PICK1 constructs in COS7 cells that lack the corresponding endogenous proteins (Fig. 3). Pearson’s correlation coefficient (PCC) analysis on immunofluorescence indicated that RAB39B and PICK1 co-localized at the perinuclear region and at the plasma membrane (flag-RAB39B/myc-PICK1: *n*=14 cells, PCC=0.70; Fig. 3b,h). As a control of specificity of RAB39B co-localization with PICK1, we co-expressed GFP-RAB39A with myc-PICK1, because RAB39A is endogenously expressed in COS7 cells. PCC analysis revealed that PICK1 highly co-localized with RAB39B but not with RAB39A (GFP-RAB39A/myc-PICK1: *n*=27 cells, PCC=0.29, adjusted *P*-value for multiple comparison (*P*-adj.)=1E−08; Supplementary Fig. 2a–c). Moreover, PCC from immunofluorescence on co-transfection of the relevant mutant constructs in COS7 cells showed that the co-localization of PICK1 and RAB39B was significantly abolished when either RAB39B is mutated in the D44F45AA (flag-RAB39B D44F45AA/myc-PICK1: *n*=14 cells, PCC=0.23, *P*-adj.=1.7E−08; Fig. 3g,h) or PICK1 is mutated in the PDZ domain (flag-RAB39B/myc-PICK1 KDAA: *n*=23 cells, PCC=0.22, *P*-adj.=1.7E−08; Fig. 3c,h).

### RAB39B–PICK1 interaction is necessary and sufficient for GluA2 trafficking

**Figure 4:**
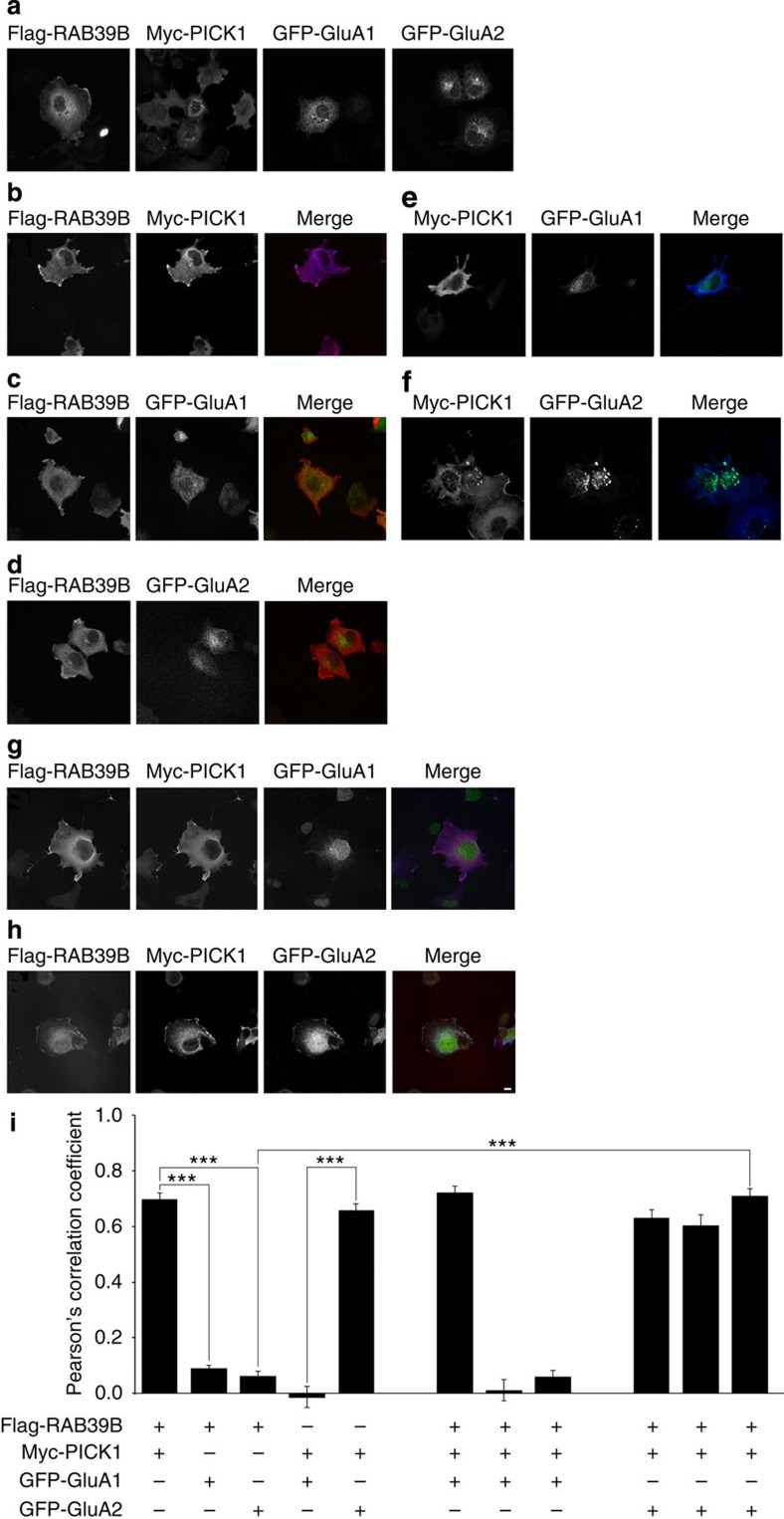
RAB39B localizes with GluA2 only in the presence of PICK1. Representative immunofluorescence images of COS7 cells (**a**) single, (**b**–**f**) double or (**g**,**h**) triple transfected with flag-RAB39B (red), myc-PICK1 (blue), GFP-GluA1 (green) or GFP-GluA2 (green). Scale bar represents 10 μm. (**i**) Histogram shows the Pearson’s correlation coefficients (PCC; means±s.e.m.) for each transfection. PCC was calculated between proteins highlighted with a bold ‘+’. Significant statistical differences were found comparing: flag-RAB39B/GFP-GluA1 PCC (*n*=26 cells; three experimental replicates; PCC=0.09, *P* adj.=1.8E−08) and flag-RAB39B/GFP-GluA2 PCC (*n*=22 cells; three experimental replicates; PCC=0.06, *P* adj.=1.8E−08) to flag-RAB39B/myc-PICK1 PCC (*n*=14 cells; three experimental replicates; PCC=0.70); myc-PICK1/GFP-GluA1 PCC (*n*=29 cells; three experimental replicates; PCC=−0.01, *P* adj.=1.8E−08) to myc-PICK1/GFP-GluA2 PCC (*n*=38 cells; three experimental replicates; PCC=0.66); flag-RAB39B/GFP-GluA2 PCC in the absence or presence of PICK1 (PCC in the presence of PICK1=0.71; *n*=12 cells; three experimental replicates; *P* adj.=1.8E−08). Benjamini–Hochberg procedure used to test statistical significance. ***P<0.001.

**Figure 5:**
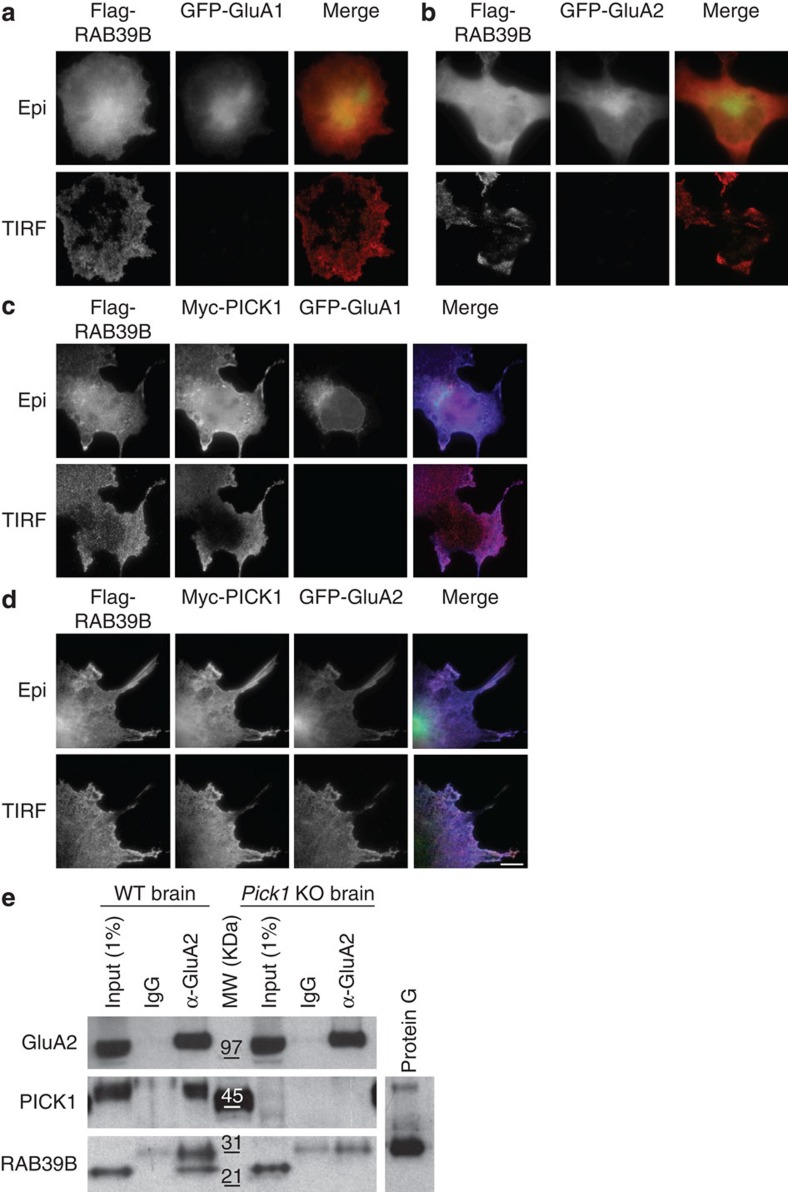
Validation of RAB39B–PICK1–GluA2 complex. Representative TIRF and epifluorescence images of COS7 cells transfected with flag-RAB39B (red) and GFP-GluA1 (green) or GFP-GluA2 (green), in the absence (**a**–**b**) or presence (**c**,**d**) of PICK1 (blue). Scale bar, 10 μm. (**e**) Representative western blots (*n*=3 experimental replicates) of immunoprecipitation from WT and *Pick1* KO mouse brain lysates (Input). The 31-kDa contaminant bands reacting with the anti-RAB39B correspond to the protein G as shown in the mock elution of protein G Sepharose-4 fast flow beads (in conditions of elution adopted as described in Methods^69^).

Given that RAB proteins direct vesicular transport^2^ and previous studies defined the functional role of PICK1 in GluA2 but not GluA1 AMPAR subunit trafficking^14^,^25^,^35^, we hypothesized that based on RAB39B and PICK1 interaction, they jointly coordinate trafficking of GluA2, but not of GluA1. To test these assumptions, we transfected into the heterologous COS7 cell system tagged constructs of RAB39B, PICK1, GluA1 and GluA2 either individually or in combination. Pearson’s correlation coefficient was measured to verify co-localization between these proteins (Fig. 4) and the receptor trafficking to the plasma membrane by total internal reflection fluorescence (TIRF) (Fig. 5).

When expressed alone, GFP-GluA1 as well as GFP-GluA2 localized at the perinuclear region (Fig. 4a). Co-expression of flag-RAB39B did not change the localization of either GFP-GluA1 or GFP-GluA2, neither RAB39B co-localized with GluA1 and GluA2 (flag-RAB39B/GFP-GluA1: *n*=26 cells, PCC=0.09, flag-RAB39B/GFP-GluA2: *n*=26 cells, PCC=0.06; Fig. 4c,d,i). Co-expression of myc-PICK1 and GFP-GluA1 did not show co-localization (myc-PICK1/GFP-GluA1: *n*=29 cells, PCC=−0.01; Fig. 4e,i). Conversely, GFP-GluA2 co-clustered with myc-PICK at the perinuclear region when co-expressed, as has been previously reported^29^ (myc-PICK1/GFP-GluA2: *n*=38 cells, PCC=0.66; Fig. 4f,i).

Remarkably, GFP-GluA2 co-localized with flag-RAB39B only when co-expressed with myc-PICK1, and a significant correlation was found between RAB39B and GluA2 depending on the presence of PICK1 in the cells (flag-RAB39B/GFP-GluA2 with PICK1: *n*=12 cells, PCC=0.71, *P*-adj.=1.8E−08 comparing flag-RAB39B/GFP-GluA2 in PICK1 absence versus presence; Fig. 4h,i). In contrast, GFP-GluA1 never showed co-localization regardless of the co-expression of RAB39B and PICK1 constructs (flag-RAB39B/GFP-GluA1 with PICK1: *n*=24 cells, PCC=0.06; Fig. 4g,i).

Next, we then demonstrated receptor trafficking to the plasma membrane by TIRF. Co-expression of flag-RAB39B did not drive the plasma membrane translocation of either GFP-GluA1 or GFP-GluA2 (Fig. 5a,b). Remarkably, in triple transfected cells, flag-RAB39B mediated the GFP-GluA2 translocation to the plasma membrane in the presence of myc-PICK1 (Fig. 5d) but not GFP-GluA1 (Fig. 5c).

All together our results clearly indicate that the effector-binding region of RAB39B interacts with the PDZ domain of PICK1, also required for interaction with the GluA2 AMPAR subunit^36^. We also demonstrated that the RAB39B−PICK1 interaction is necessary for the RAB39B-driven trafficking of GluA2 cargo. PICK1-mediated functional linkage between RAB39B and GluA2-containing AMPAR relies on PICK1 dimer to act as a scaffold that hosts the other two proteins. In fact, GluA2 co-immunoprecipitates with PICK1 and RAB39B in a wild -type mouse brain lysate, but not in the *Pick1*-knockout brain, demonstrating the existence of the endogenous complex and that PICK1 acts as the bridge between RAB39B and GluA2 (Fig. 5e).

### A model of interactions among the RAB39B-PICK1-GluA2

**Figure 6:**
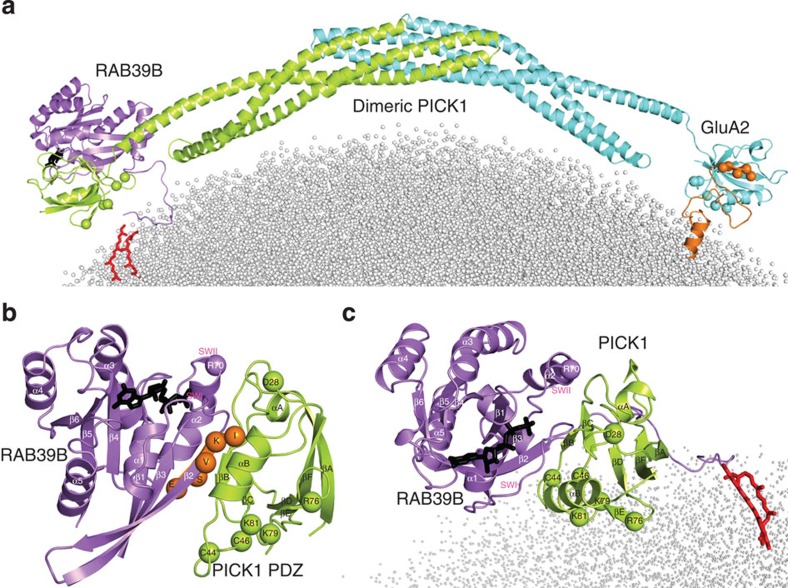
Dimeric PICK1 acts as a scaffold for RAB39B and GluA2. (**a**) A zoom into the predicted RAB39B:2PICK1:GluA2Ct complex is shown on a liposome. (**b**,**c**) A zoom into the PICK1 PDZ-RAB39B interface is shown. The GluA2Ct extracted from the other PDZ subunit, following the Cα-atom superimposition of the two PDZ domains, is shown, represented as orange spheres. The spheres of the interacting D28 from PICK1 PDZ and R70 from RAB39B are shown as well. (**c**) An alternative view of the PICK1 PDZ-RAB39B complex is shown highlighting the orientation of the lipid-binding motifs on PDZ and of the two geranylgeranyl molecules on the two C-terminal cysteines of RAB39B with respect to the surface of the liposome.

To gain insight, at the atomic level, into the supramolecular organization of RAB39B, PICK1 and GluA2 AMPAR subunit, we modelled the structure of RAB39B:2PICK1:GluA2 complex (Fig. 6a and Supplementary Methods). This required comparative modelling and rigid-body docking to predict structure and interaction modes of PICK1 and RAB39B (see Methods). In contrast, the complex between the GluA2 C-terminus (GluA2Ct; aa 858-ESVKI-862) and PICK1 was extracted from a conformational ensemble solved by NMR^37^. In our structural model, the N-terminal half of the β2-strand of RAB39B (that is, amino-acid stretch 43–47 of the effector-binding region) interacts with the PDZ domain of PICK1 in a similar and partially overlapping manner as GluA2Ct, which is a β-strand as well (Fig. 6a–c). The major portions of RAB39B participating in the interface with PICK1 PDZ include (a) the 43–47 segment of the β2-strand, which makes inter-backbone and inter-side chain interactions with PICK1 βB and with αB; (b) switch 1 (swI) that interacts with the PICK1 βA/βB loop and αB; and (c) the switch 2 (swII) that interacts with PICK1 βC as well as the βA/βB and βB/αA loops (b and c). The predicted complex highlights D44 and F45 in the N-terminal half of the β2-strand as playing a central role in the interface, D44 making a salt bridge with K83 of PICK1 PDZ. Such a centrality of D44 and F45 has been validated by *in vitro* experiments (Fig. 3). Remarkably, the PICK1 βA/βB loop of PDZ holds the K27-D28 pair that we found essential for PICK1−RAB39B interaction. Structure predictions indicate D28 as more important than K27 in RAB39B recognition. Indeed, K27 is buried at the βB/αB interface, its positively charged nitrogen atom being about 9 Å from the carboxylate oxygen atoms of D38 on the swI of RAB39B, and we postulate that the effect of the K27A mutation on RAB39B recognition, if any, is indirect. In contrast, D28 recognizes the G protein via a salt bridge with R70 in swII. Structure prediction therefore suggests that the interactions of RAB39B and GluA2 with the PDZ domain of PICK1 are mutually exclusive. In line with evidence that PICK1 can dimerize^33^, the structural model suggests that dimerization of PICK1 is a prerequisite for simultaneous recognition of both RAB39B and GluA2 each by one of the PICK1 molecules in the PICK1 dimer (Fig. 6a–c). The existence of such complex is supported by our co-immunoprecipitation experiments shown above.

### RAB39B directs GluA2 trafficking in neurons

The two most prevalent AMPAR variants contain a tandem of either GluA1 and GluA2 or GluA2 and GluA3 subunits^19^,^20^,^21^. Exports of GluA1/GluA2 hetero-tetramers from the ER requires GluA1 interaction with SAP97 (refs 23, 24), while GluA2/GluA3 hetero-tetramers need GluA2 interaction with PICK1 (ref. 25).

To assess whether the RAB39B-PICK1 tandem directs trafficking of GluA2/GluA3 hetero-tetramers in their endogenous neuronal context, we first analysed the intracellular localization of RAB39B and PICK1 in flag-RAB39B-transfected primary mouse hippocampal neurons (Supplementary Fig. 3). Flag-RAB39B appears to partially co-localize with PICK1 in the cell body, instead of along dendrites (Supplementary Fig. 3a). In a similar manner, flag-RAB39B and PICK1 co-localize in part with the ER or Golgi markers, calreticulin and GM130, respectively (Supplementary Fig. 3b,c). No co-localization was observed between flag-RAB39B and adaptor protein 2 (AP2), which was used as a negative control^10^. Although RAB39B and PICK1 are certainly involved in many other intracellular pathways, our data support that RAB39B–PICK1 interaction has a role in cargo trafficking between the ER and the Golgi compartments.

We then analyse the effect of *Rab39b* downregulation in transduced murine primary hippocampal neurons with previously described shRab39b and shScramble lentiviral particles^10^. Silencing with shRab39b led to a 40% downregulation of RAB39B expression levels (mean±s.e.m., shScramble: 1.1±0.13, shRab39b: 0.68±0.08; *n*=3; *t*-test *P*=0.035), without affecting protein expression levels of PICK1, GluA1, GluA2 and GluA3 (Supplementary Fig. 4).

**Figure 7:**
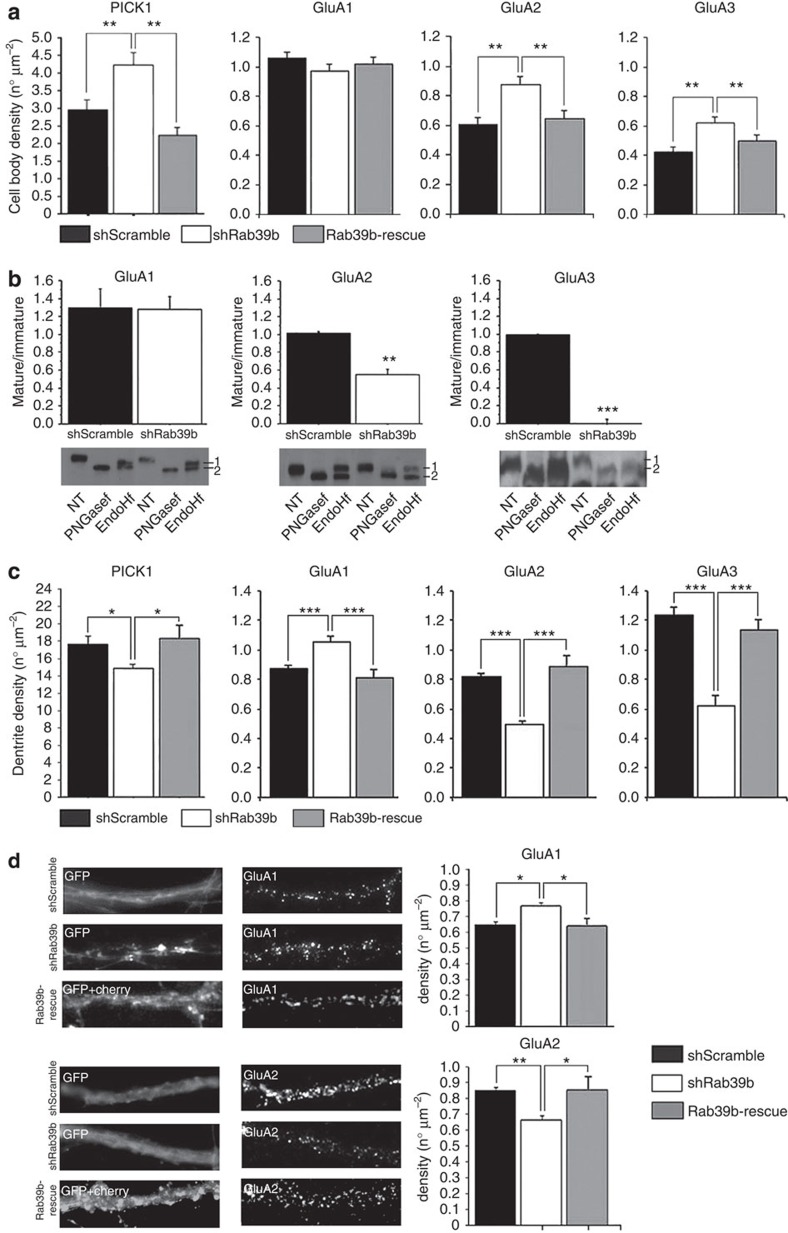
PICK1, AMPARs neuronal localization, maturation and GluA2 surface expression. (**a**) Quantification of PICK1 (shScramble *n*=21 cells; shRab39b *n*=21 cells; Rab39b-rescue *n*=8 cells; Student’s *t*-test shScramble versus shRab39b *P*=0.009; Rab39b-rescue versus shRab39b *P*=0.002), GluA1 (shScramble *n*=10 cells; shRab39b *n*=10 cells; Rab39b-rescue *n*=9 cells), GluA2 (shScramble *n*=9 cells; shRab39b *n*=5 cells; Rab39b-rescue *n*=11 cells; Student’s *t*-test shScramble versus shRab39b *P*=0.003; Rab39b-rescue versus shRab39b *P*=0.004) and GluA3 (shScramble *n*=6 cells; shRab39b *n*=7 cells; Rab39b-rescue *n*=9 cells; Student’s *t*-test shScramble versus shRab39b *P*=0.03; Rab39b-rescue versus shRab39b *P*=0.04) cell body density in shScramble-, shRab39b- and Rab39b-rescue-treated mouse hippocampal neurons. (**b**) Quantification of the ratio between mature **(1)** and immature **(2)** forms of AMPARs in shRab39b- compared with shScramble-treated neurons after PNGasef or EndoHf digestion. NT: non-treated neurons. Representative western blots (lower panels) showing the maturation ratio for GluA1 (*n*=3 experimental replicates), GluA2 (*n*=3 experimental replicates; Student’s *t*-test *P*=0.002) and GluA3 (*n*=3 experimental replicates; Student’s *t*-test *P*=1.28E−04). (**c**) Quantification of PICK1 (shScramble *n*=14 cells; shRab39b *n*=15 cells; Rab39b-rescue *n*=8 cells; Student’s *t*-test shScramble versus shRab39b *P*=0.03; Rab39b-rescue versus shRab39b *P*=0.01), GluA1 (shScramble *n*=10 cells; shRab39b *n*=10 cells; Rab39b-rescue *n*=9 cells; Student’s *t*-test shScramble versus shRab39b *P*=7.4E−04; Rab39b-rescue versus shRab39b *P*=8.1E−04), GluA2 (shScramble *n*=10 cells; shRab39b *n*=5 cells; Rab39b-rescue *n*=9 cells; Student’s *t*-test shScramble versus shRab39b *P*=2.8E−07; Rab39b-rescue versus shRab39b *P*=3.1E−05) and GluA3 (shScramble *n*=6 cells; shRab39b *n*=7 cells; Rab39b-rescue *n*=9 cells; Student’s *t*-test shScramble versus shRab39b *P*=1.5E−05; Rab39b-rescue versus shRab39b *P*=4.7E−05) dendrite density in shScramble-, shRab39b- and Rab39b-rescue-treated mouse hippocampal neurons. (**d**) Representative images of shRab39b, shScramble and Rab39b-rescue neurons immunostained without permeabilization for the extracellular N-terminal region of GluA1 and GluA2. Quantification of positive puncta at cell surface shows a significant increase of GluA1 (shScramble *n*=41 cells; shRab39b *n*=40 cells; Rab39b-rescue *n*=10; Student’s *t*-test shScramble versus shRab39b *P*=0.03; Rab39b-rescue versus shRab39b *P*=0.006) and significant decrease of GluA2 (shScramble *n*=89 cells; shRab39b *n*=70 cells; Rab39b-rescue *n*=8; Student’s *t*-test shScramble versus shRab39b *P*=0.006; Rab39b-rescue versus shRab39b *P*=0.01) subunits. The number of cells belongs from a minimum of three experimental replicates. *P<0.05; **P<0.01; ***P<0.001.

We first determined the effect of RAB39B silencing on the intracellular localization of PICK1 GluA1, GluA2 and GluA3 in the murine hippocampal neurons by immunofluorescence at 14 DIV. We found an increase in PICK1, GluA2 and GluA3 levels in the cell body of shRab39b-treated neurons as compared with the neurons treated with the control shRNA (mean±s.e.m., PICK1 shScramble *n*=21: 2.98±0.28, shRab39b *n*=21: 4.25±0.35; *t*-test *P*=0.009; GluA2 shScramble *n*=9: 0.60±0.05, shRab39b *n*=5: 0.88±0.05; *t*-test *P*=0.003; GluA3 shScramble *n*=6: 0.42±0.03, shRab39b *n*=7: 0.62±0.04; *t*-test *P*=0.005; Fig. 7a and Supplementary Fig. 5a), which suggests that there is a defect in protein trafficking when levels of RAB39B are downregulated.

Next, we analysed the effect of RAB39B silencing on the intracellular trafficking in murine hippocampal neurons of the AMPAR subunits GluA1, GluA2 and GuA3 by examining their glycosylation status. Both AMPAR subunits are *N*-glycosylated and their N-glycans are subsequently modified in the ER before they are targeted to the plasma membrane. We therefore deglycosylated lysates from RAB39B silenced or control neurons with either Endo-β-*N*-acetylglucosaminidase H (EndoH), which removes only unmodified N-glycans^25^, or, as a control, with peptide-*N*-Glycosidase F (PNGasef), which removes all N-glycans, before analysis of the AMPAR subunits by immunoblotting. In the control lysates two isoforms, differing in their mobility in gel, were detected for GluA1, GluA2 and GluA3 on treatment with EndoH: an upper band, corresponding to the mature isoform (as it acquired partial EndoH resistance), and a lower band, corresponding to the immature isoform (as it acquired no EndoH resistance). The ratio between the two bands thus is a measure for AMPAR subunit maturation. As such, we determined that GluA2 and GluA3 maturation was impaired in shRab39b-treated neurons compared with the control neurons (mean±s.e.m., GluA2 shScramble: 0.96±0.05, shRab39b: 0.52±0.05; *n*=3; *t*-test *P*=0.002; GluA3 shScramble: 1.0±0.07, shRab39b: 0.0±0.0; *n*=3; *t*-test *P*=0.0001; Fig. 7b), while GluA1 maturation was not affected by the shRab39b silencing (mean±s.e.m., shScramble: 1.29±0.21, shRab39b: 1.28±0.15; *n*=3; *t*-test *P*=0.9; Fig. 7b).

We next examined the role of the RAB39B-PICK1 tandem in post-Golgi trafficking of AMPAR subunits along dendrites. We noted that, in RAB39B-silenced cells, the increased PICK1 as well as GluA2 and GluA3 density at the cell body (Fig. 7a and Supplementary Fig. 5a) was mirrored by a decreased PICK1, GluA2 and GluA3 density along dendrites (mean±s.e.m., PICK1 shScramble *n*=14: 17.82±1.13, shRab39b *n*=15: 14.98±0.61; *t*-test *P*=0.03; GluA2 shScramble *n*=10: 0.82±0.02, shRab39b *n*=5: 0.49±0.02; *t*-test *P*=2.8E−7; GluA3 shScramble *n*=7: 1.24±0.06, shRab39b *n*=10: 0.62±0.07; *t*-test *P*=1.6E−5; Fig. 7c and Supplementary Fig. 5b). Likewise, downregulation of RAB39B resulted in a decrease in surface density of GluA2 as measured by immunostaining without permeabilization of the cells (mean±s.e.m., GluA2 shScramble *n*=89: 0.88±0.02, shRab39b *n*=70: 0.72±0.03; *t*-test *P*=0.006). Curiously, surface expression of GluA1 slightly increased on RAB39B silencing (mean±s.e.m., shScramble *n*=41: 0.62±0.02, shRab39b *n*=40: 0.77±0.02; *t*-test *P*=0.03; Fig. 7d), mirrored by a significant increase along dendrites (mean±s.e.m., GluA1 shScramble *n*=10: 0.87±0.02, shRab39b *n*=10: 1.05±0.04; *t*-test *P*=0.0007; Fig. 7c and Supplementary Fig. 5b). The reintroduction of RAB39B by co-transducing shRab39b-treated neurons with a cherryRab39b-rescue lentiviral particles recovered the intracellular distribution of PICK1 (shRab39b versus Rab39b-rescue *n*=8; *t*-test *P*=0.002), GluA2 (shRab39b versus Rab39b-rescue *n*=11; *t*-test *P*=0.004) and GluA3 (shRab39b versus Rab39b-rescue *n*=12; *t*-test *P*=0.007) and GluA1 (shRab39b versus Rab39b-rescue *n*=10; *t*-test *P*=0.006) and GluA2 (shRab39b versus Rab39b-rescue *n*=8; *t*-test *P*=0.01) surface expression (Fig. 7a,c,d and Supplementary Fig. 5a,b).

Finally, downregulation of RAB39B has a mild effect on spine morphology without altering spine number and type (mean±s.e.m., spine length shScramble *n*=26: 1.20±0.02, shRab39b *n*=30: 1.12±0.02; *t*-test *P*=0.05; spine width shScramble *n*=25: 0.68±0.01, shRab39b *n*=29: 0.64±0.009; *t*-test *P*=0.02; Supplementary Fig. 5c).

These results indicate that in neurons the RAB39B-PICK1 tandem directs trafficking specifically of GluA2/GluA3 heterotetramers to the Golgi and, consequently, regulates GluA2 cell surface expression and spine morphology.

### RAB39B determines AMPAR composition and synaptic transmission

**Figure 8:**
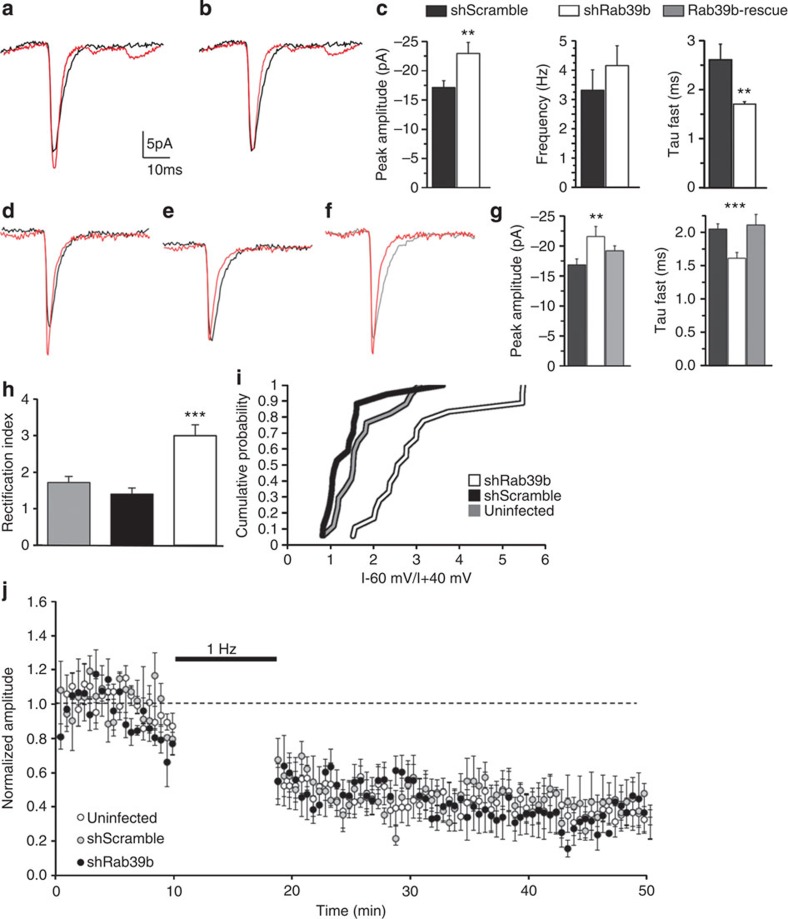
Synaptic transmission and AMPAR composition. (**a**) Averages of 100 mEPSCs from shScramble (black trace) and shRab39b (red trace) superimposed showing the peak amplitude increase in silenced neurons (**c**). In (**b**) the same traces are superimposed and the silenced average mEPSCs (red) is normalized to the peak of the shScramble (black), revealing the lower decay kinetic of shRab39b currents (**c**). (**c**) Histograms show the average changes in the mEPSCs peak amplitude, frequency and decay kinetics (shScramble *n*=14 cells and shRab39b *n*=13 from three experimental replicates; Student’s *t*-test *P*<0.01). (**d**) Averages of 100 mEPSCs from shScramble (black) and shRab39b (red) superimposed as in (**a**) and (**e**) as in (**b**). The grey trace in (**f**) is the average of 100 mEPSCs from Rab39b-rescue neurons, in which the average peak amplitude and kinetics resembles that of the shScramble values (peak amplitude −19.2±0.8 pA; tau 2.11±0.21 ms, *n*=10 cells). These results are summarized in the histograms in (**g**). The number of cells belongs from a minimum of three experimental replicates. (**e**) Rectification index histogram from uninfected, shScrambled and shRab39b-treated organotypic slices (*n*=6 experimental replicates for each condition) and (**f**) cumulative probability indicate a highly significant increase of the rectification index in *Rab39b* silenced neurons (Student’s *t*-test *P*<0,0001 shRab39b versus shScramble; *P*=0,0007 shRab39b versus uninfected; *P*=0,19 uninfected versus shScramble). (**g**) LTD representation of uninfected, shScrambled and shRab39b-treated organotypic slices. **P<0.01; ***P<0.001.

Since our results so far demonstrated that RAB39B is key to ensure efficient trafficking of GluA2/GluA3 to the Golgi compartment, and, hence, its cell surface expression, we reasoned that ultimately RAB39B controls the availability of GluA2 and thus AMPAR composition at the post-synapses. Because the subunit composition, in turn, determines Ca^2+^ permeability of the AMPARs and therefore impacts on synaptic transmission, we asked whether RAB39B-PICK-directed trafficking of GluA2/GluA3 is key for glutamatergic synapses. To this end, we performed electrophysiological recordings on primary murine hippocampal neurons that were infected with the shRab39b or control shScramble lentiviruses at low doses to ensure that most pre-synaptic inputs would come from non-infected (unaltered) neurons, while post-synaptic outputs would come from infected (silenced) neurons (Supplementary Fig. 6a,b). The amplitude of miniature EPSCs was slightly but significantly larger in RAB39B-silenced neurons (mean±s.e.m. in pA, shScramble *n*=14: −17.1±1.1, shRab39b *n*=13: −22.9±1.9; *t*-test *P*<0.01) (Fig. 8a–c). The miniature frequency, however, was unaffected, suggesting that indeed RAB39B silencing occurred mostly in post-synaptic neurons due to the low-density lentiviral infection (Fig. 8c). Interestingly, kinetic analysis of the miniature currents indicated that RAB39B silencing accelerated decay (mean±s.e.m. in ms, shScramble *n*=14: 2.60±0.32, shRab39b *n*=13: 1.71±0.05; *t*-test *P*<0.01) but not rise times of synaptic currents (Fig. 8b,c). The specificity of shRab39b effect was determined using a rescue experiment. Electrophysiological recordings were carried out on primary murine hippocampal neurons that were infected with the shRab39b or control shScramble lentiviruses at multiplicity of infection (MOI) 1 to ensure that pre-synaptic inputs and post-synaptic outputs come from infected (silenced) neurons and after 6 days in culture transfected with a flag-RAB39B-rescue, as previously described^10^ (Supplementary Fig. 6c,d). The reintroduction of RAB39B recovered the amplitude and kinetic of the miniature currents (Fig. 8d–g).

Since RAB39B directs GluA2 traffic and thus cell surface expression, we attributed the electrophysiological changes on RAB39B silencing to an altered subunit composition of synaptic AMPARs. To test this hypothesis, we monitored inward rectification of AMPAR-mediated synaptic responses in organotypic hippocampal slices from mice that were injected in the CA1 with the relevant lentiviruses (shScramble or shRab39b) by evoked EPSCs, recorded at −60 mV and +40 mV. The ratio of synaptic responses at +40 mV over responses at −60 mV was calculated to serve as a rectification index (R.I.). We found a significant increase in the R.I. of RAB39B silenced neurons (mean±s.e.m., uninfected *n*=6: 1.801±0.175; shScramble *n*=6: 1.403±0.166; shRab39b *n*=6: 2.998±0.297; ANOVA: *P*<0.0001) (Fig. 8h,i), indicative of enrichment at synapses of inward rectifying AMPARs. Since GluA2-lacking AMPARs in fact are inward rectifying^38^ we concluded that the alterations in synaptic transmission are due to a lowered GluA2 availability. This interpretation also fits with our observation that RAB39B down regulation leads to increased miniature EPSC amplitude (Fig. 8a–g), in agreement with the higher conductance of GluA2-lacking AMPARs^39^. Finally, we tested whether long-term depression (LTD) was altered in RAB39B down regulated cells, given the involvement of PICK1 in activity-dependent AMPAR endocytosis and LTD^15^,^40^. To this end, we assessed NMDAR-dependent LTD in shRab39b-, shScramble- and non-infected CA1 neurons (Fig. 8j). After LTD induction (500 stimuli at 1 Hz, with postsynaptic depolarization at −40 mV), AMPAR-mediated synaptic responses were equally depressed in the three conditions. These results suggest that, in contrast with PICK1, RAB39B is not required for LTD. Therefore, RAB39B does not appear to be involved in activity-dependent AMPAR endocytosis or recycling.

## Discussion

Our results define RAB39B in tandem with its downstream effector PICK1 as a key molecule dedicated to GluA2 AMPAR subunit trafficking. We demonstrate a molecular complex where the dimerization of PICK1 is a prerequisite for simultaneous recognition of both RAB39B and GluA2 by each one of the PDZ domains of PICK1, in a PICK1 dimer conformation.

**Figure 9:**
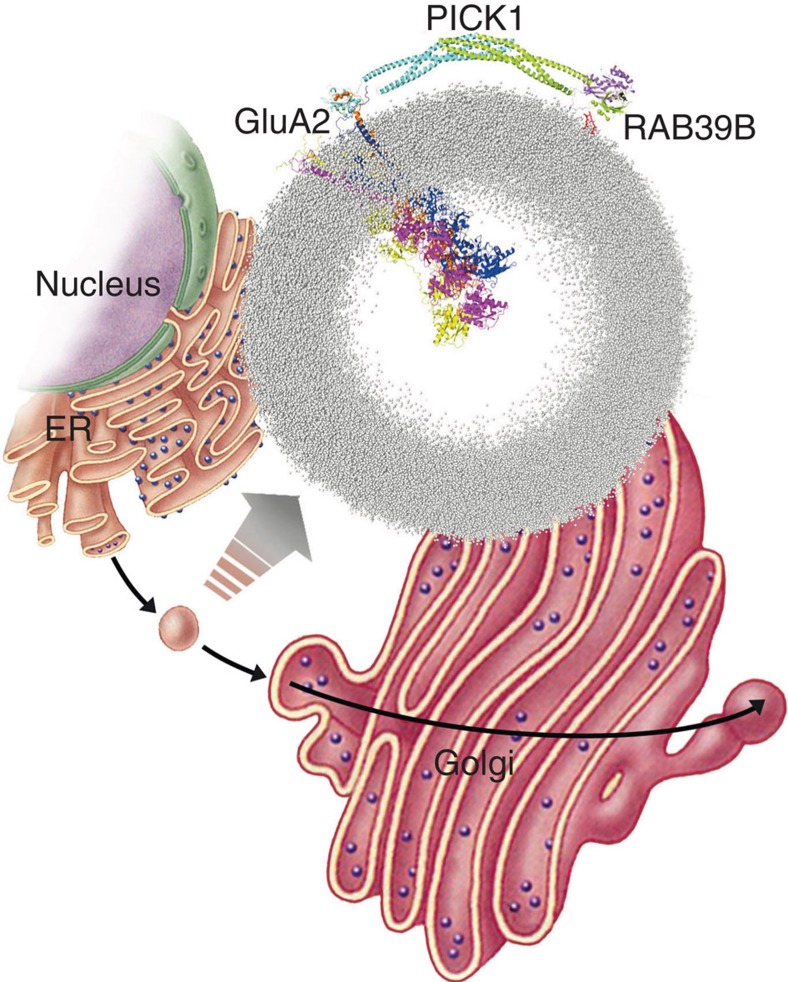
RAB39B-PICK1-GluA2 model acting at ER–Golgi interface. Proposed model of RAB39B-PICK1-GluA2 complex on a 1,2-dioleoyl-sn-glycero-3-phosphocholine (DOPC) coarse grained liposome model with a 40 nm diameter (grey dots are the atoms of DOPC) emphasizing RAB39B-driven traffic of GluA2 cargo between the ER and Golgi compartment.

Subsequently, the remarkable inference from this study is that RAB39B interacting with PICK1 ensures selectively the GluA2/GluA3 AMPAR exit from the ER and its maturation entering into the *cis*-Golgi (Fig. 9). Indeed, despite the demonstrated relevance of regulators of AMPAR surface expression, trafficking and post-synaptic targeting and human memory formation and maintenance (for example, KIBRA^41^ and TMS4SF2 (ref. 42)), only few proteins that are involved in the trafficking between the ER and the Golgi of AMPAR subunits are known. Exit of plasma membrane proteins from the ER poses a rate-limiting step and is subjected to tight control, to safeguard that only properly folded and assembled complexes leave the ER and are transported to the *cis*-Golgi^43^,^44^, to finally reach the plasma membrane. However, rules governing subunit assembly and the progression of distinct AMPAR complexes through the secretory pathway are largely unknown. It is known that AMPAR subunits preferentially homodimerize soon after the translocation in the ER and tetramerize before ER exit in GluA1/GluA2 and GluA2/GluA3 complexes^25^. Greger and coauthors^25^ found unexpected differences in the subcellular localization and ER export kinetics of the GluA1 and GluA2 subunits. Whereas GluA1 receptor exit the ER and traffic to the surface, GluA2 partly remains in the ER, suggesting the existence of an intracellular GluA2 reserve pool. GluA2 ER pool is, at least partly, associated with GluA3, whereas GluA1/A2 heteromers are mostly confined to post-ER compartments. Finally, to become competent for ER export, AMPARs need to co-assemble with auxiliary proteins. In the brain, auxiliary subunits of the stargazing/TARP (transmembrane AMPAR regulatory protein) family and the cornichon family are physically associated with the channel and regulate their trafficking and gating^45^,^46^,^47^,^48^,^49^. Recently, Cornichon-2/-3 was described as cargo receptors to selectively bind GluA1, and not GluA2, in hippocampal neurons, allowing the forward trafficking of GluA1-containing AMPARs from the ER to Golgi and finally their synaptic expression^50^. Instead, our study identifies RAB39B as the first RAB GTPase regulating a vesicular trafficking step for the transport of GluA2/GluA3 heteromers into the secretory pathway.

Finally, our analysis of the steady-state levels of AMPAR subunits at the post-synaptic membrane of *Rab39b* silencing hippocampal neurons demonstrates that the alteration in the RAB39B-mediated secretory pathway leads to a decrease in surface GluA2 density and an increase in GluA1 AMPAR subunits. This observation was confirmed functionally by the analysis of the rectification properties of AMPAR-mediated synaptic transmission resulting in an increased rectification index suggestive of an increased fraction of GluA2-lacking AMPARs at synapses related to the increased amplitude of miniature EPSCs. Because it was previously described that, in the hippocampus, heterotetramers of GluA1/GluA2 and GluA2/GluA3 subunits, together with a smaller contribution from GluA1 homomers, represent the most common combinations in excitatory synapses^22^, we could speculate that altering RAB39B-mediated early trafficking step of GluA2/GluA3, the result will be an increase in GluA1 Ca^2+^-permeable homomers.

In fact, the AMPA-type glutamate receptors play a critical role in synaptic plasticity underlying learning and memory by mediating the majority of fast excitatory synaptic transmission and by trafficking into and out of the synapse^51^. The presence of GluA2 in heteromeric AMPAR renders the channel impermeable to Ca^2+^ and Zn^2+^, thus influencing channel kinetic, channel conductance and synaptic transmission. In contrast, GluA2-lacking AMPAR is Ca^2+^-permeable and is mostly observed in young animals or after an epoch of plasticity-inducing neuronal activity^52^,^53^,^54^. In fact, our results describe a slight alteration in spine morphology, depicting a scenario of immature spines in shRab39b-treated neurons, as they are shorter and wider than control neurons. Previous studies described that Ca^2+^-permeable AMPARs are important in experience-dependent and synaptic plasticity^55^. These studies suggest that Ca^2+^-permeable AMPAR play a prominent role in maintaining circuits in a labile state where further plasticity can occur, thus promoting metaplasticity^56^. Moreover, it was shown that the abnormal expression of Ca^2+^-permeable AMPARs is implicated in drug addiction and memory disorders, with significant implication in the development of therapeutic approaches to these disorders^56^. In addition, alteration of the relative proportion of AMPAR subunits after pathological insults^54^,^57^ or in response to physiological stimuli^58^ will shift the balance towards homomers and in turn alter the signalling landscape of the neuron.

Altogether our findings shed light on the molecular mechanisms responsible for the maturation and the trafficking of GluA2/GluA3 AMPAR in the secretory pathway, and pave the way to explain the functional role of RAB39B in cognitive disorder.

Unsolved questions remain still open. At first, what other molecules convey the transport competence to RAB39B? One possibility could be the involvement of additional downstream effectors proteins that give the directionality and the timing for RAB39B–PICK1–GluA2 complex movement. GM130 a *cis*-Golgi protein involved in accepting vesicles from the ER^59^,^60^, as well as dynein/dynactin motor complex, driving microtubule-dependent trafficking from the ER to Golgi^61^ and along dendrites to the post-synaptic sites^62^ or myosin Va^63^ involved in dendritic and post-synaptic actin-dependent trafficking^64^ could be the most reliable factors. At present, RAB39B is required for the transport of GluA2-containing AMPAR from the ER to the Golgi, but we cannot assess its involvement from the Golgi to the neuronal surface. Future studies will undoubtedly be necessary to answer these questions as well as the availability of *Rab39b*-null mouse model to better define the link between RAB39B-mediated intracellular trafficking and cognitive disorders.

## Methods

Most of the experiments were on mouse hippocampal neurons prepared from mouse embryos at E18 days. Astroglial feeder layer cultures were prepared from mouse cortices at post-natal day 2. Mice were C57Bl/6N genetic background and were obtained from Charles River, Italy. Mice were euthanized in accordance with ‘Institutional Animal Care and Use Committee San Raffaele (IACUC)’ at San Raffaele Scientific Institute, Milan, Italy and approved by the Italian National Ministry of Health, IACUC ID 470 and in accordance with the guidelines established by the European Community Council Directive of 24 November 1986 on the use of animals in research (86/609/EEC).

### Plasmids generation

pGEX-RAB5 and pGEM-p110β were a gift from Professor M. Zerial (Max Planck, Dresden, Germany). GST-RAB39A was a gift from Professor M. Fukuda (Tohoku Univ., Japan). Myc-PICK1, pGEX-PICK1, myc-PDZ PICK1, flag-Δ121 PICK1, myc-KDAA PICK1, myc-K83A PICK1, GFP-GluA1 and GFP-GluA2 were a gift from Dr M. Passafaro (CNR Institute of Neuroscience, Milan, Italy) and Professor R. Huganir (Johns Hopkins University, Baltimore, MD, USA). pGEM-PICK1 was created amplifying the full-length rat *Pick1* coding sequence using specific primers (PICK1 For: 5′-GCGCCACCATGTTTGCAGACTTAGACTATGACAT-3′; PICK1 Rev: 5′-TCAGGAGTCACACCAGCTTCCG-3′). In the forward primer the KOZAK sequence (CCACC) was inserted. The PCR product was then cloned into pGEM-T-easy (Promega). Flag-RAB39B was created as previously described^10^. pGEX-RAB39B was created amplifying the mouse *Rab39b* cDNA from flag-RAB39B vector using specific primers (pPC86 For: 5′-GCGGTCGACCATGGAGGCCATCTGGCTGTACC-3′; pGEX Rev: 5′-GCGGCGGCCGCCTAGCACAAACATCTCCTCTCTGA-3′), and the PCR product was than cloned in frame into SalI and NotI sites of p-GEX-4T-2 plasmid (GE Healthcare Life Sciences).

The site-directed mutagenesis using the Quick Change Lightning kit (Stratagene) was used to generate nucleotide deletion or mutations on flag-RAB39B or pGEX-RAB39B to obtain the following plasmids: flag-ΔER (deletion of 45 nucleotides from +105 to +147; deletion of 15 amino acids from +35 to +49), flag-D38A (mutations on nucleotides from +112 to +114, mutation on amino acid +38, D38A), flag-V41G42V43AAA (mutations on nucleotides from +121 to +129, mutations on amino acids from +41 to +43, V41A, G42A, V43A), flag-D44F45AA (mutations on nucleotides from +130 to +135, mutations on amino acids from +44 to +45, D44A, F45A), pGEX-RAB39B S22N (mutations on nucleotides from +63 to +65, mutation on amino acid +22, S22N), pGEX-RAB39B Q68L (mutations on nucleotides from +64 to +66, mutation on amino acid +22, Q68L). pDBleu-RAB39B was created amplifying the human *RAB39B* cDNA, obtained from retro-transcription of linfoblastoid cells RNA from control patients, using specific primers (pPC86 For: 5′-GCGGTCGACCATGGAGGCCATCTGGCTGTACC-3′; pPC86 Rev: 5′-GCGGCGGCCGCTCTAGCACAAACATCTCCTCTCTGA-3′). The PCR product was than cloned in frame into SalI and NotI sites of pDB-Leu (ProQuest Two-hybrid system, Gibco BRL). FLAG-RAB39B-rescue, cherryRAB39B-rescue and GFP-RAB39A was created as previously described^10^.

### Yeast two-hybrid assay

Yeast two hybrid screening was performed following the manufacturer’s instruction (ProQuest Two-hybrid system, Gibco BRL). The full-length human *RAB39B* cDNA was cloned into the pDBLeu vector, in frame with the GAL4-binding domain, and used as bait to screen a human foetal brain cDNA library (ProQuest Pre-made cDNA Libraries) cloned into the pPC86 vector in frame with the GAL4-activating domain. Positive colonies grew on plates containing 10 mM 3-amino-1,2,4-triazole(3-AT) without tryptophan and leucine and expressed different reporter genes: HIS3 and URA3 allowed the growth, respectively, in medium lacking histidine and uracil, LacZ induced a colorimetric reaction in the presence of 5-bromo-4-chloro-3-indolyl-β-D-galactopyranoside (X-gal). cDNA plasmids from positive clones were recovered via TOP10F’ *E. coli*, plated on ampicillin and sequenced. Yeast two-hybrid screening was repeated two times and only positive colonies expressing three reporter genes (HIS3, LacZ and URA3) were taken in consideration. The test was performed following the manufacturer’s instructions (ProQuest Two-hybrid system, Gibco BRL).

### GST pull-down

GST fusion proteins were prepared in *E. coli* strain BL21DE3, isolated and immobilized on glutathione-Sepharose 4B beads (GE Healthcare; as decribed^65^). GST pull-down experiments were carried out with mouse brain lysates or COS7 cells transfected with different constructs or *in vitro* transcribed and translated PICK1.

A mouse brain was lysed with HKT buffer (4 mM Hepes pH 7.4, 400 mM KCl, 4 mM EDTA, 2% Triton X-100, Protease Inhibitor cocktail) and rotated at 4 °C for 45 min before dilution 1:2 with HKT buffer and centrifuging at 16,000 *g* for 10 min to pellet debris, or it was lysed with RIPA buffer (500 mM TrisHCl pH 7.4, 200 mM NaCl, 1 mM EDTA, 1% Triton X-100, 1% NP-40, Protease Inhibitor cocktail) and rotated at 4 °C for 1 h before centrifuging at 500 *g* for 10 min to pellet debris.

COS7 cells were transfected with plasmids expressing different PICK1 constructs as myc-PDZ, flag-Δ121, myc-KDAA, myc-K83A, or different RAB39B constructs as flag-RAB39B, flag-ΔER, flag-D38A, flag-V41G42V43AAA, flag-D44F45AA. After 2 days of transfection, COS7 cells were lysed in RIPA buffer, rotated at 4 °C for 1 h before centrifuging at 12,500 *g* for 20 min to pellet debris and sonicated for 5 min.

PICK1 was *in vitro* transcribed and translated modifying the standard protocol (GeHealthcare) as follows: the *in vitro* reaction was reduced to 30 min instead of 90 min.

All the lysates were pre-cleared with 120 μg GST immobilized on glutathione-Sepharose 4B beads (GE Healthcare) for 1 h at 4 °C.

Forty micrograms of GST-RAB39B in presence of GDP or GTPγS (Sigma-Aldrich) or 40 μg of GST-RAB39B S22N and GST-RAB39B Q68L were incubated on rotation 3 h at 4 °C with 8 mg of mouse brain lysed in HKT or RIPA buffer, or 2 mg of COS7 cells expressing myc-PDZ, flag-Δ121, myc-KDAA, myc-K83A or 10 μl of the *in vitro* translational reaction of PICK1. Eighty micrograms of GST-PICK1 immobilized on glutathione-Sepharose 4B beads were incubated on rotation 3 h at 4 °C with 16 mg of mouse brain lysed in HKT buffer or 2 mg of COS7 cells expressing flag-RAB39B or flag-RAB39B ΔER, flag-D38A, flag-V41G42V43AAA or flag-D44F45AA.

In all the experiments, GST alone and/or GST-RAB5 GDP and GTPγS immobilized on glutathione-Sepharose 4B beads were used as control.

After washing, the proteins are eluted with elution buffer (25 mM Reduced Glutathione, 500 mM Tris pH 8, 300 mM NaCl, Protease Inhibitor cocktail) for 30 min at RT, resuspended in 5 × SDS sample buffer (4% SDS, 1.3 M Sucrose, 5 mM EDTA, 10% β-mercaptoethanol, 0.5 M Tris pH 6.8), analysed by SDS–PAGE and western blot with appropriate antibodies: the anti-PICK1 polyclonal antibody (Abcam, #ab3420, 1:500 WB), the anti-PKCα polyclonal antibody (Cell Signaling, #2056, 1:1,000 WB), anti-p110β polyclonal antibody (Santa Cruz, #sc-602, 1:200 WB), the anti-RAB39B polyclonal antibody (produced by BioGenes against the hypervariable C-terminal RAB39B sequence CVVHSSEEVIKSERR and tested for binding specificity as showed in Supplementary Fig. 1d; 1:500 WB), anti-myc monoclonal antibody (Sigma-Aldrich; #M4439; 1:5,000 WB) and the anti-FLAG polyclonal antibody (Sigma-Aldrich, #F1804, 1:1,000 WB), anti-GluA2 monoclonal antibody (Millipore, #MAB397, 1:200 IF), anti-RAB5 monoclonal antibody (BD Transduction, #610282, 1:500 WB) and anti-GST polyclonal antibody (Santa Cruz, #sc-459, 1:500 WB). Band intensity was measured with ImageJ.

### Immunoprecipitation

Immunoprecipitation studies were carried out with lysates prepared from HEK293T cells co-expressing flag-RAB39B and myc-PICK1 or from wild-type mouse brain or *Pick1*-knockout mouse brain. Transfected cells were washed in phosphate-buffered saline (PBS) Ca^2+^/Mg^2+^ (0.1 mM CaCl_2_, 1 mM MgCl_2_) and scraped in CHAPS buffer (1% CHAPS; Sigma-Aldrich; 0.1 mM EDTA pH 8, Protease Inhibitor Cocktail in PBS solution) and rotated at 4 °C for 1 h before centrifuging at 13,200 *g* for 20 min to pellet debris. Mouse brains were lysed in HBST buffer (10 mM HEPES pH 7.4, 150 mM NaCl, 0.5% Triton X-100, Protease Inhibitor Cocktail in H_2_O solution), homogenized 10 times by hands, rotated at 4 °C for 10 min and centrifuged at 13,500 *g* for 20 min at +4 °C to pellet debris.

All the lysates were pre-cleared with washed protein G-Sepharose-4 fast flow beads (GE Healthcare/Amersham Biosciences) for 2 h at 4 °C.

Immunoprecipitation studies were performed, in presence of 3 mM GTPγS (Sigma-Aldrich), incubating HEK293T cells transfected with flag-RAB39B and myc-PICK1 with an anti-myc monoclonal antibody (6 μg; Sigma-Aldrich; #M4439) for 3 h at 4 °C on rotation, and wild type or *Pick1* KO mouse brain lysates with an anti-GluA2 monoclonal antibody (5 μg; Millipore; #MAB397) 24 h at 4 °C on rotation. IgG (5 μg) was used as a control.

The beads were eluted in 5 × SDS sample buffer (4% SDS, 1.3 M Sucrose, 5 mM EDTA, 10% β-mercaptoethanol, 0.5 M Tris pH 6.8) 10 min at room temperature and analysed by 4–12% polyacrylamide gel on SDS–PAGE and western blot with appropriate antibodies: anti-myc monoclonal antibody (Covance, #MMS-150P, 1:5,000 WB), anti-FLAG polyclonal antibody (Sigma-Aldrich, #F7425, 1:1,000 WB), anti-GluA2 polyclonal antibody (SySy, #182103, 1:1,000 WB), anti-PICK1 polyclonal antibody (JH2906 was made into the laboratory of Prof. R. Huganir Johns Hopkins University, Baltimore, MD, USA, 1:250 WB), anti-RAB39B polyclonal antibody (produced by BioGenes against the hypervariable C-terminal RAB39B sequence CVVHSSEEVIKSERR; 1:500 WB).

### Cell cultures, transfection and lentiviral transduction

COS7 and HEK293T cells were grown in Dulbecco’s modified Eagle’s medium (Gibco) supplemented with 10% fetal bovine serum (Gibco), 1% glutamine, 1% penicillin and streptomycin and were regularly passaged to maintain exponential growth. COS7 cells were transfected using Lipofectamine 2000 (Invitrogen) following the manufacturer’s instructions and fixed 24 h after transfection.

Mouse hippocampal neurons were transduced (MOI1) at DIV1 with lentiviral particles expressing shScramble or shRab39b as previously described^10^. See Supplementary Experimental Procedures for details.

### Deglycosilation

Instructions for the use of EndoHf and PNGasef (NEB) were followed according to the manufacturer’s instructions to digest proteins, with minor modifications. In brief, cultured hippocampal neurons transduced with shRab39b or shScramble were lysed with Lysis Buffer (1% SDS, 2 mM EDTA, 10 mM hepes pH 7,4, Protease Inhibitor Cocktail). A total of 260 μg of lysed were for denatured 15 min at 55 °C and digested with EndoHf or PNGasef for 2 h at 37 °C. SDS loading buffer (5 × ) was finally added to samples, which were split in three different parts, 30, 30 and 200 μg, and analysed by western blot with 6% SDS–PAGE. GluA1, GluA2 and GluA3 were detected using a anti-GluA1 polyclonal antibody (SySy, #182003, 1:1,000 WB), anti-GluA2 polyclonal antibody (SySy; #182103, 1:1,000 WB) and anti-GluA3 polyclonal antibody (made into the laboratory of Professor R. Huganir Johns Hopkins University, Baltimore, MD, USA, 1:1,000 WB).

### Immunofluorescence

Standard immunofluorescence experiments were carried out by fixing the cells for 15 min with 4% paraformaldehyde, 4% sucrose (Sigma-Aldrich) in 120 mM sodium phosphate buffer, pH 7.4. Coverslips were rinsed three times with PBS and then incubated overnight at 4 °C into a humidified chamber with the primary antibody (anti-flag polyclonal antibody: Sigma-Aldrich; poly #F7425, 1:200 IF, anti-flag monoclonal antibody: Sigma-Aldrich, #F1804, 1:400 IF; anti-myc monoclonal antibody: Sigma-Aldrich; #M4439; 1:5,000 WB and 1:400 IF; anti-PICK1: Thermo Scientific, #PA1-073, 1:100 IF; anti-calreticulin: Sigma, #C4606, 1:500 IF; anti-GM130: BD Transduction, #610823, 1:50 IF) appropriately diluted in goat serum dilution buffer (GSDB; 15% goat serum, 450 mM NaCl, 0.3% Triton X-100, 20 mM sodium phosphate buffer, pH 7.4). Coverslips were washed three times within 30 min with high salt buffer (HS: 500 mM NaCl, 20 mM sodium phosphate buffer, pH 7.4) and then incubated with the appropriate secondary antibodies (Molecular Probes, Invitrogen) for 90 min at room temperature. After three washes with HS over 30 min and one wash with 5 mM sodium phosphate buffer, pH 7.4, and coverslips were mounted with Vectashield (Vectalab).

Visualization of intracellular endogenous AMPAR subunits in neurons was done by hiding the portion of receptors exposed on the membrane surface with primary monoclonal antibodies recognizing the N-terminal portion (anti-GluA1: Millipore, #MAB2263, 1: 1:200 IF; anti-GluA2: Millipore, #MAB397, 1:200 IF; anti-GluA3: made into the laboratory of Prof. R. Huganir Johns Hopkins University, Baltimore, MD, USA, 1:100 IF) diluted in GSDB without Triton X-100 as well as the HRP-conjugated secondary antibody (Biorad, 1:200), and then cells were incubated with the primary monoclonal antibodies recognizing the N-terminal portion diluted in GSDB with 0.3% Triton X-100 as well as the appropriate secondary antibody (Molecular Probes, Invitrogen).

To visualize GluA1 and GluA2 exposed on the cell surface, the primary monoclonal antibodies recognizing the N-terminal portion were diluted in GSDB without Triton X-100, as well as the secondary antibody (Molecular Probes, Invitrogen).

### Image acquisition and analysis

For Western blots, ImageJ Analysis Software (‘Analyze gels’ plugin) was used; full scan of western blots is shown in Supplementary Fig. 6. For GST pull-down experiments, PICK1 binding to GST-RAB39B GDP versus GST-RAB39B GTPγS is quantified as follow: signal from GST-RAB39B GTPγS binding protein is normalized on the 50 kDa GST signal and set at 100%; consequently, the level of GST-RAB39B GDP binding protein, normalized on the 50 KDa GST band, is expressed as percentage of 100.

Confocal images of COS7 cells were obtained using Leica SP8 SMD laser scanning confocal. For spine morphology fluorescent images were acquired with a Biorad MRC1024 confocal microscope, using a Nikon 60 × objective with sequential acquisition setting at 1,280 × 1,024 pixel resolution and image data were a *z* series projection of about 5–10 images, each averaged 4 times and taken at 0.7 μm depth intervals.

Pictures of immunostained hippocampal neurons were captured with the same exposure conditions using DeltaVision microscope (Applied Precision) equipped with a × 60 or × 100 objectives. At least 10 Z-space slices of 0.20 μm were deconvolved and flattened by maximum projection.

TIRF images, setting 110 nm as the distance from the coverslip, were acquired using Leica SR GSD 3D TIRF microscope (Leica).

Pearson’s correlation coefficient was calculated using Volocity software to measure the correlation between two proteins.

Morphometric spine measurements were made with NeuronStudio software.

ImageJ Analysis Software (‘Gran filter’ plugin setting the size from 1 to infinity) was used to measure AMPAR subunits and PICK1 density relatively to the area of infected cell body and dendrites.

### Electrophysiology in cultured hippocampal neurons

Seventeen to twenty DIV hippocampal neurons transduced at low or high MOI with shScramble or shRab39b, and in some case transfected with Rab39b as rescue, were patched and whole-cell recordings were made on infected neurons held at −70 mV in the presence of 3 μM TTX. mEPSCs were acquired until the cell was stable and analysed using pClamp10 (Molecular Devices). Threshold mEPSC amplitude was set at 5 pA, and 300–500 events were collected and averaged to calculate the mean mEPSC amplitude, frequency and kinetics for each culture preparation examined. See Supplementary Experimental Procedures for details.

### Electrophysiology in organotypic hippocampal slices

Hippocampal slice cultures were prepared from postnatal day 5–7 mice C57BL/6N. After 1 day in culture every slice was transduced with lentiviral particles (shScramble or shRab39b). After 10–12 days in culture, simultaneous voltage-clamp whole-cell recordings were obtained from nearby infected and uninfected CA1 pyramidal neurons. Whole-cell recordings were made with a Multiclamp 700B amplifier (Axon Instruments) and electrophysiological data were collected with pCLAMP software (Molecular Devices). See Supplementary Experimental Procedures for details.

### Statistical analysis

Data are expressed as mean±s.e.m. Statistical significance was assessed using paired Student’s *t*-test as appropriate (for two groups comparison). We verified the difference between groups by means of t-type test statistic and exact *P* values computed with permutation methods to avoid any distributional assumption or asymptotical approximation. *P* values were, then, adjusted for multiplicity applying the Benjamini–Hochberg procedure (false discovery rate)^66^.

### Molecular modelling

The structural models of PICK1 and RAB39B were achieved by comparative modelling (that is, by MODELLER^67^), whereas the interaction between them was predicted by protein docking (that is, by ZDOCK^68^). The CTD of GluA2 was built by comparative modelling as well. Details of Computational modelling are provided as Supplementary Information.

## Author contributions

M.L.M., M.G. and P.D. designed the experiments. M.G. performed cell biology experiments and with L.M. the electrophysiological experiments under the conceptual advise of M.P. and J.A.E. M.L.M. performed two-hybrid screening, biochemical, cell biology experiments and A.G. helped M.L.M. F.F. supervised RAB39B modelling and docking simulations, modelled PICK1, built the RAB39B:2PICK1:AMPAR complex. F.R. modelled RAB39B and did docking simulations and analyses. S.B., H.F., M.A. and R.H. gave conceptual advices to M.L.M. for biochemical experiments. S.G. gave conceptual advice, supervised the RPF fellowship from the industrial point of view and contributed to the manuscript. F.F., E.V.A., J.A.E., R.H. and P.D. wrote the manuscript. P.D. supervised the project.

## Additional information

**How to cite this article:** Mignogna, M. L. *et al.* The intellectual disability protein RAB39B regulates selectively GluA2 trafficking determining synaptic AMPAR composition. *Nat. Commun.* 6:6504 doi: 10.1038/ncomms7504 (2015).

## Supplementary Material

Supplementary Information Supplementary Figures 1-6, Supplementary Methods and Supplementary References
