# Supplementary material for: The intellectual disability protein RAB39B selectively regulates GluA2 trafficking to determine synaptic AMPAR composition
Source: Nat Commun. 2015 Mar 18;6:6504. doi: 10.1038/ncomms7504 (PMC4383008; doi:10.1038/ncomms7504)
Supplement: Supplementary Information — Supplementary Figures 1-6, Supplementary Methods and Supplementary References [file ncomms7504-s1.pdf]

Supplementary Figures

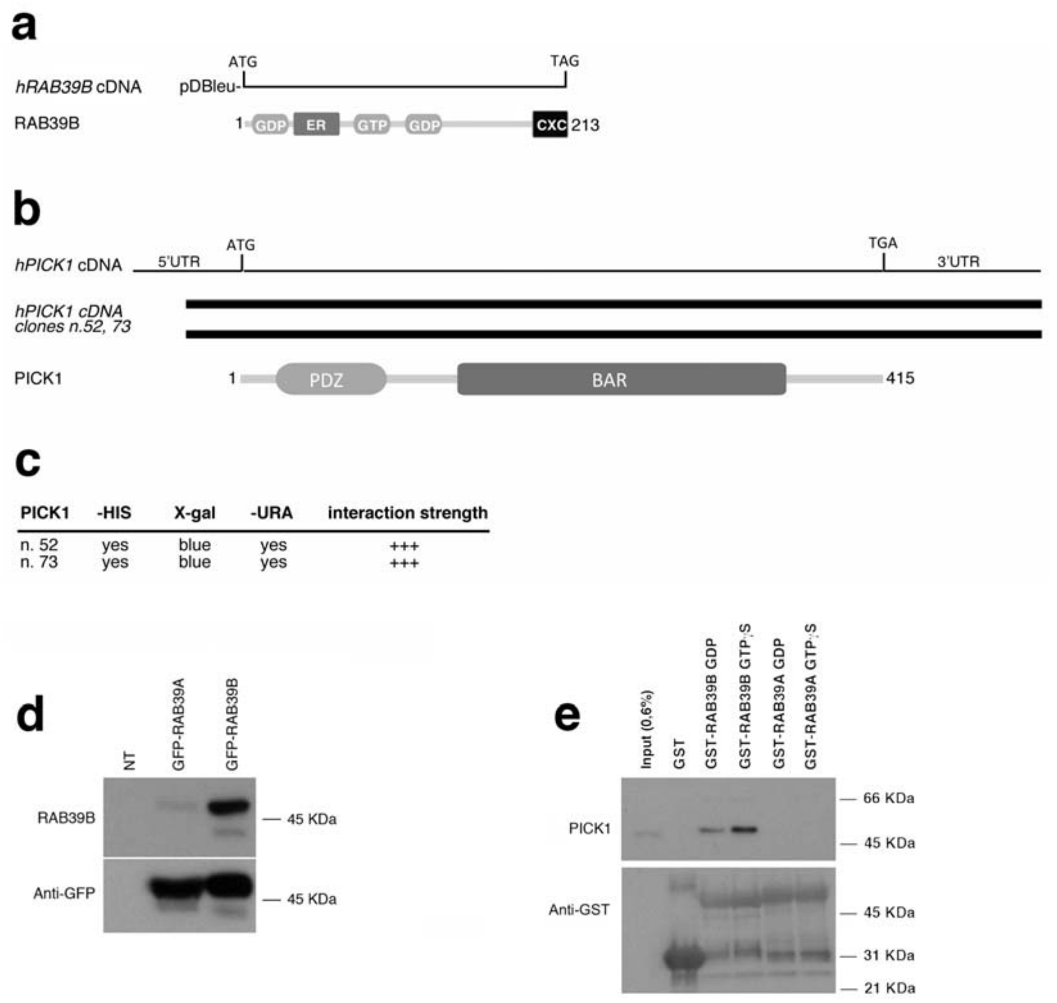

Supplementary Figure 1. Two-hybrid screening, anti-RAB39B specificity and GST-RAB39A pull down. (a) Open reading frame of human *RAB39B* cDNA cloned into pDB-Leu and used as bait in two-hybrid assay. **(b)** Representation of full-length human *PICK1* cDNA, the corresponding cDNA sequence of clones n. 52 and 73 (black lines) identified in the two-hybrid screening, encoding for the entire PICK1 protein. **(c)** Summary of interaction strengths between RAB39B and clones n. 52 and 73. Each "+" indicates the ability to activate HIS3, lacZ and URA3 reporter genes. **(d)** Representative western blots (n = 3 experimental replicates) of COS7 cells transfected with GFP-RAB39B or GFP-RAB39A immunoblotted with anti-RAB39B or anti-GFP. NT: non-transfected cells. **(e)** GST-RAB39A and GST-RAB39B pull down experiments (n = 3 experimental replicates) immunoblotted with PICK1 and anti-GST.

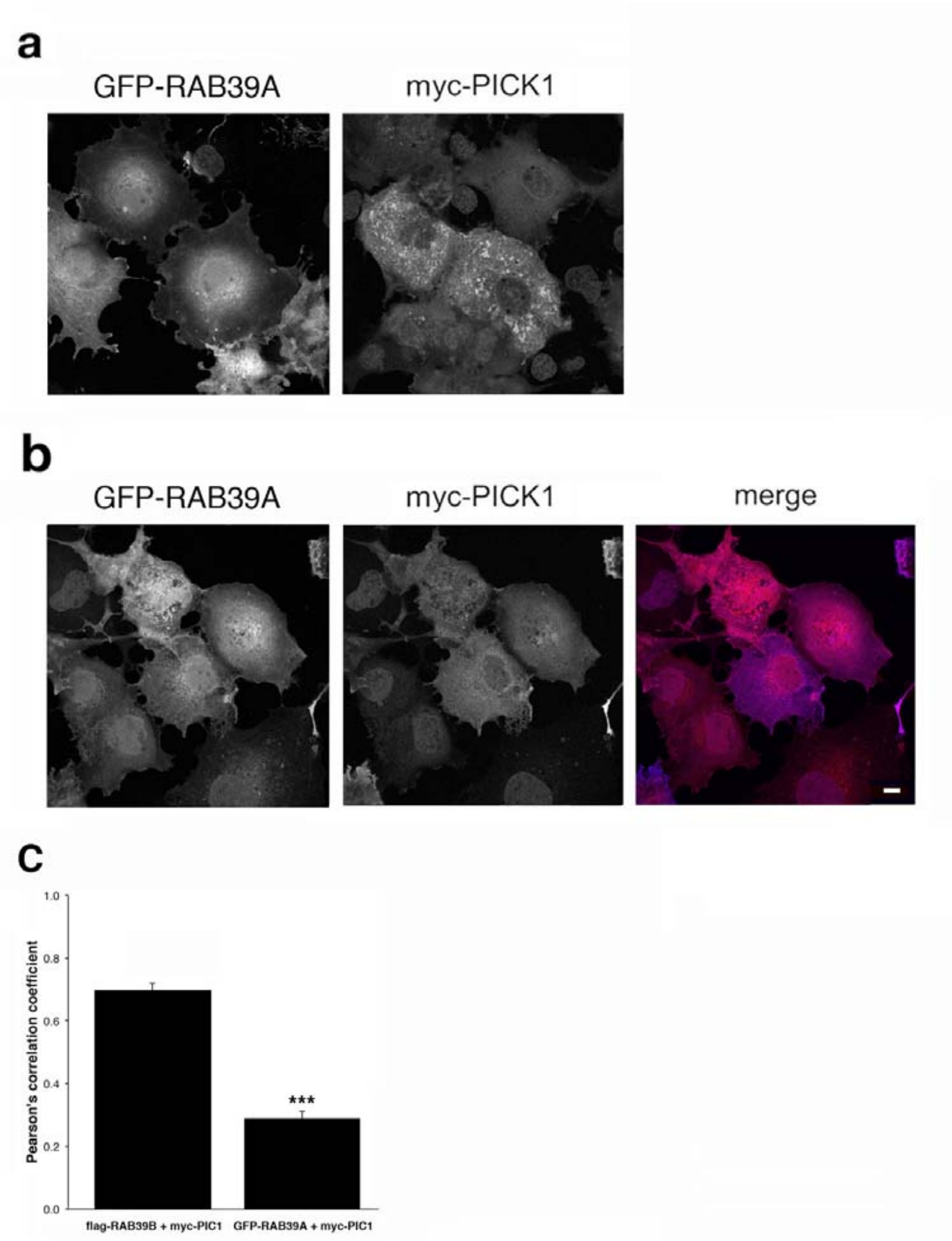

Supplementary Figure 2. RAB39A does not interact with PICK1. Representative images of COS7 cells **(a)** single or **(b)** double transfected with GFP-RAB39A (red) and myc-PICK1 (blue) constructs. **(c)** Pearson's Correlation Coefficient calculated between GFP-RAB39A/myc-PICK1 and compared to flag-RAB39B/myc-PICK1 (GFP-RAB39A/myc-PICK1 n = 27 cells, PCC = 0.29; flag-RAB39B/myc-PICK1 n = 14 cells, PCC = 0.70, p-adj.= 1E-08). Scale bar represents 10 μm. Number of cells belongs from 3 experimental replicates.

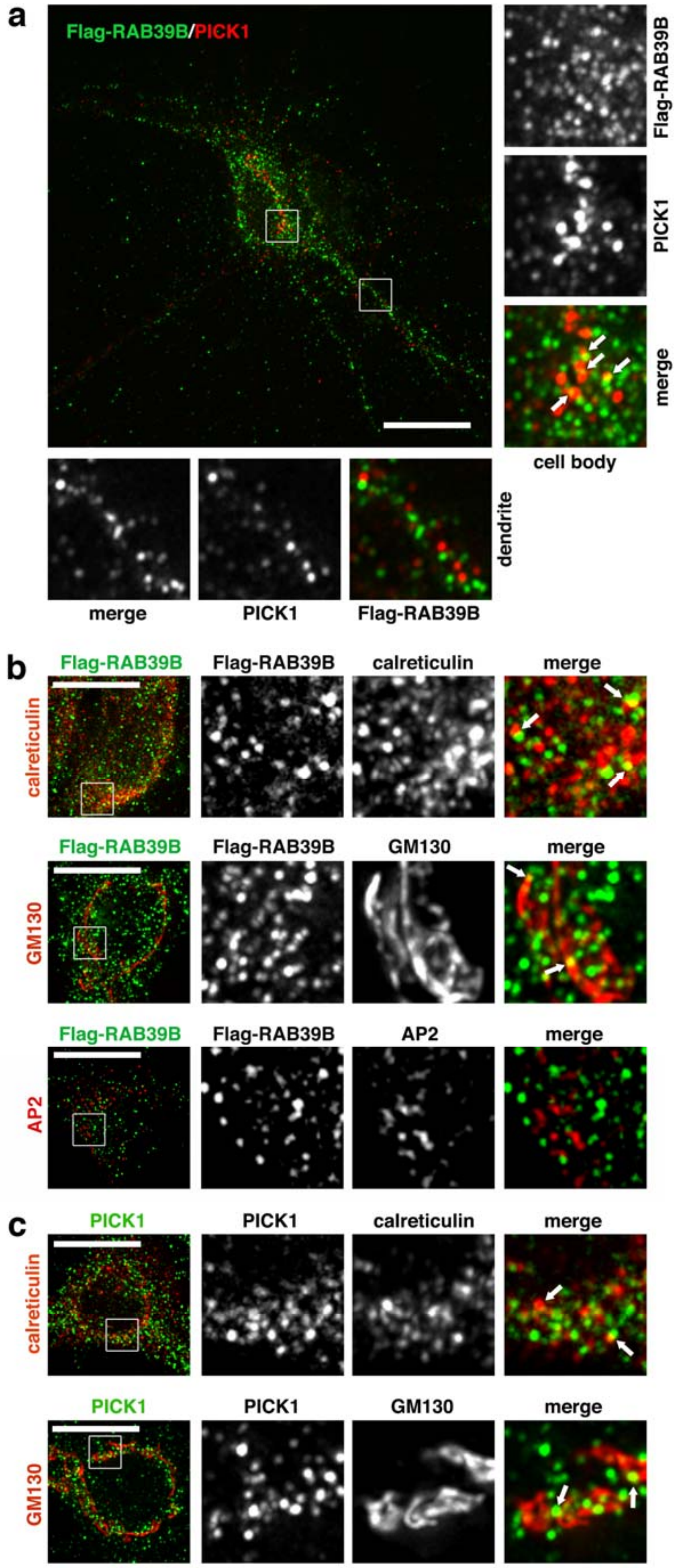

Supplementary Figure 3. Neuronal localization of RAB39B and PICK1 with ER and Golgi markers.

Representative immunofluorescence images of mouse hippocampal neurons stained for flag-RAB39B, PICK1,

calreticulin, GM130 and AP2 adaptor protein 2. **(a)** Cells were transfected with flag-RAB39B and stained with anti-flag (green) and PICK1 (red). Empty white squares in **(a)** highlighted cell body and dendrite magnification showed in the right part or below the principal image. White arrows indicated flag-RAB39B and PICK1 co-localized puncta. **(b)** Double immunofluorescence between flag-RAB39B (green) and calreticulin (red, ER) or GM130 (red, Golgi) or AP2 (red) markers. **(c)** Double immunofluorescence between PICK1 (green) and calreticulin (red, ER) or GM130 (red, Golgi) markers. Empty white squares in the principal images in **(b and c)** highlighted cell body magnification showed in the right part and white arrows indicate co-localization spots. Scale bar represents 10 µm.

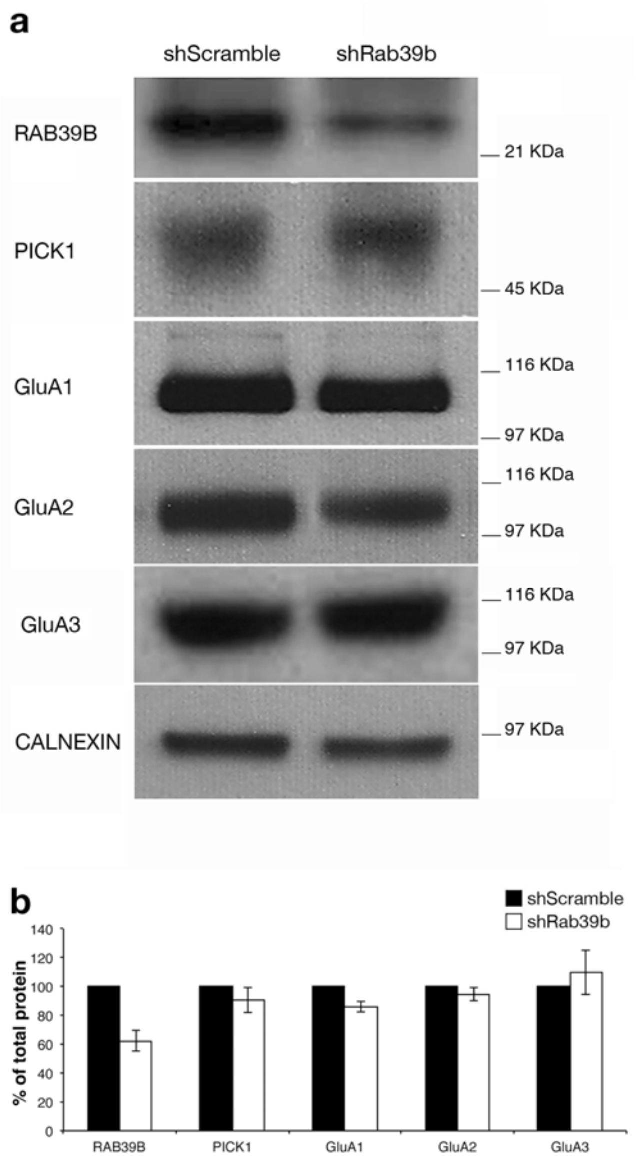

Supplementary Figure 4. Protein amount of PICK1 and AMPAR subunits under RAB39B down regulation. Representative western blots (n = 3 experimental replicates) of lysates from 14 DIV mouse hippocampal neurons transduced with lentiviral particles expressing either shScramble or shRab39b. Calnexin serves as loading reference. **(b)** Percentage (± s.e.m.) of total protein normalized on the intensity of calnexin band.

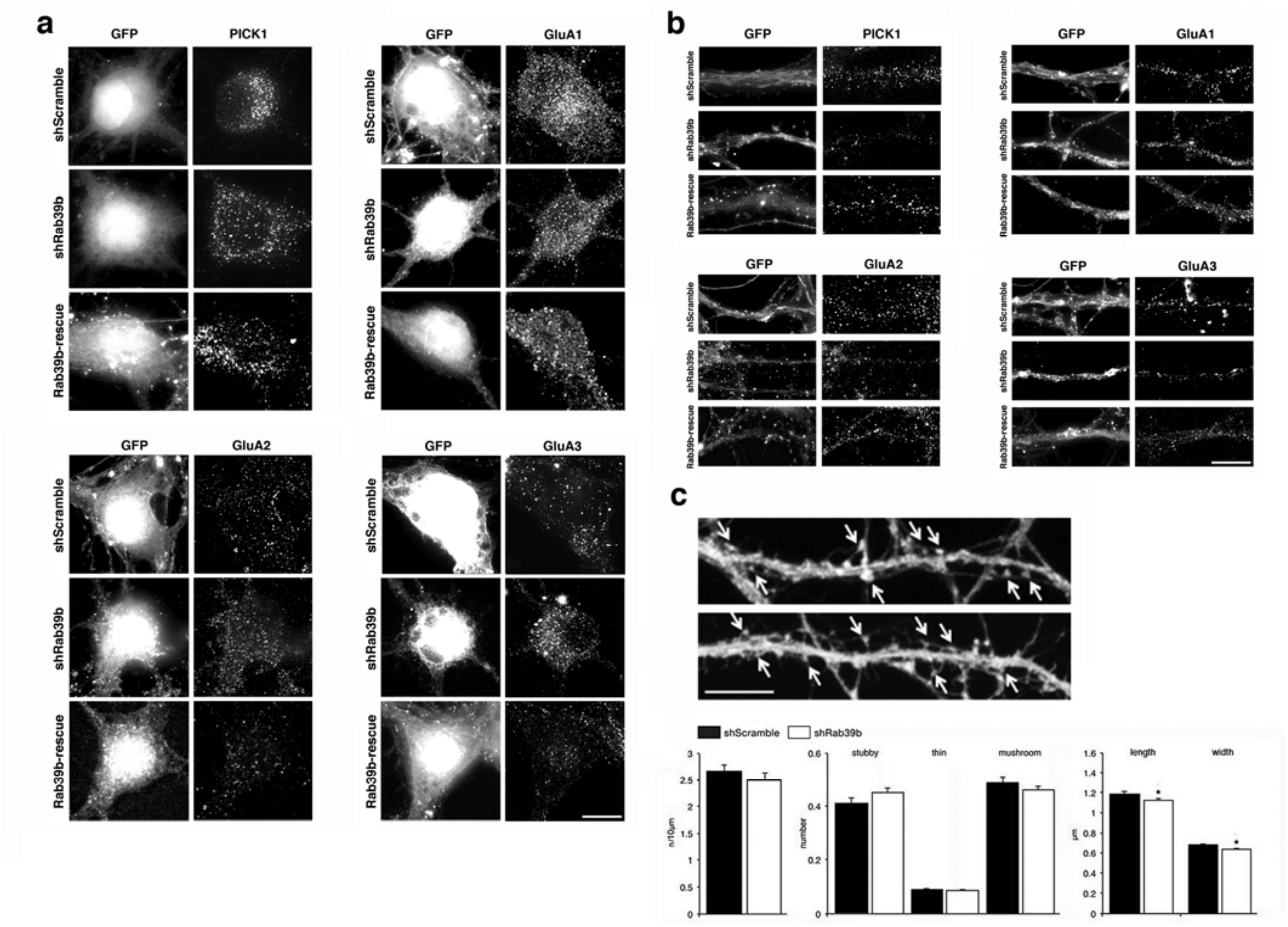

Supplementary Figure 5. PICK1, AMPARs intracellular localization and spine morphology. (a-b) Representative immunofluorescence images performed on 14 DIV primary hippocampal neurons transduced with shScramble, shRab39b and Rab39b-rescue. Images show PICK1, GluA1, GluA2 and GluA3 in the cell body **(a)** and in dendrites **(b)**. **(c)** Representative images show a mild change in spine morphology (spines are indicated by arrows, upper panel) in shRab39b-treated neurons. Lower panel represent the quantification of spine number, type and morphology: spines result shorter (shScramble n = 26 cells; shRab39b n = 30 cells; Student's t-test p = 0.05) and wider (shScramble n = 25; shRab39b n = 29; Student's t-test p = 0.02). Number of cells belongs from 3 experimental replicates. Scale bar represents 10 μm. *p < 0.05.

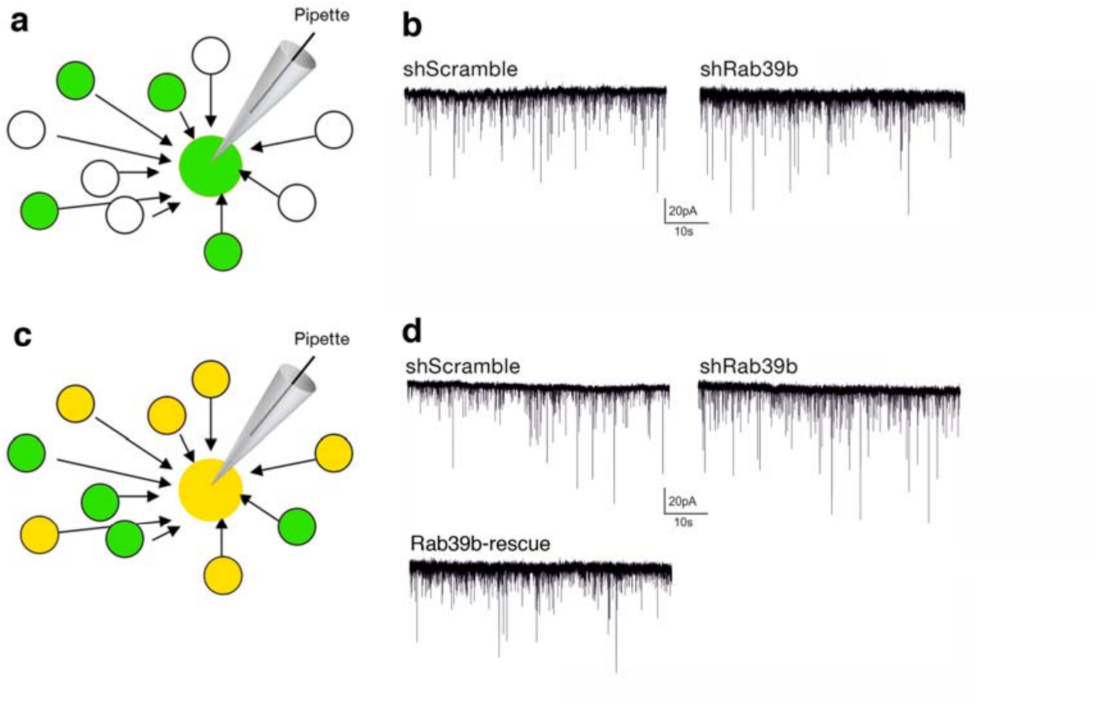

Supplementary Figure 6. The scheme in **(a)** represents the probable situation with low infection dose, recording from a postsynaptic infected neuron (green) with a majority of not-infected presynaptic contacts (white). The corresponding examples of 1 minute mEPSCs recordings (Vh=-70mV and 30μM TTX) is shown in **(b)**, for shScramble and shRab39b neurons. The scheme in **(c)** represents the high infection level condition, together with the transfection of Rab39b (flag-Rab39b-rescue, yellow). The corresponding 1 minute mEPSCs recordings (as in **b**) is reported in **(d)**, for shScramble, shRab39b and Rab39b-rescue.

Fig.1a

Fig.1b

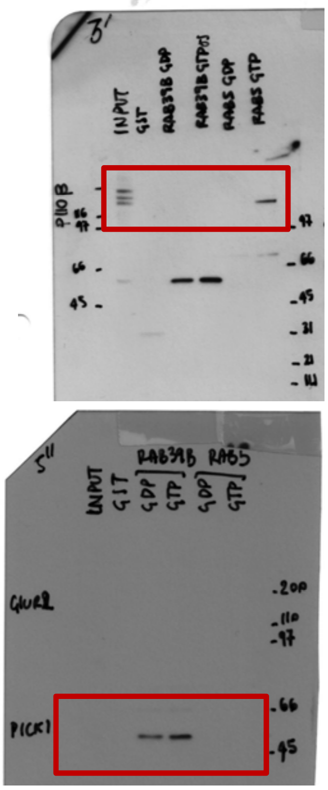

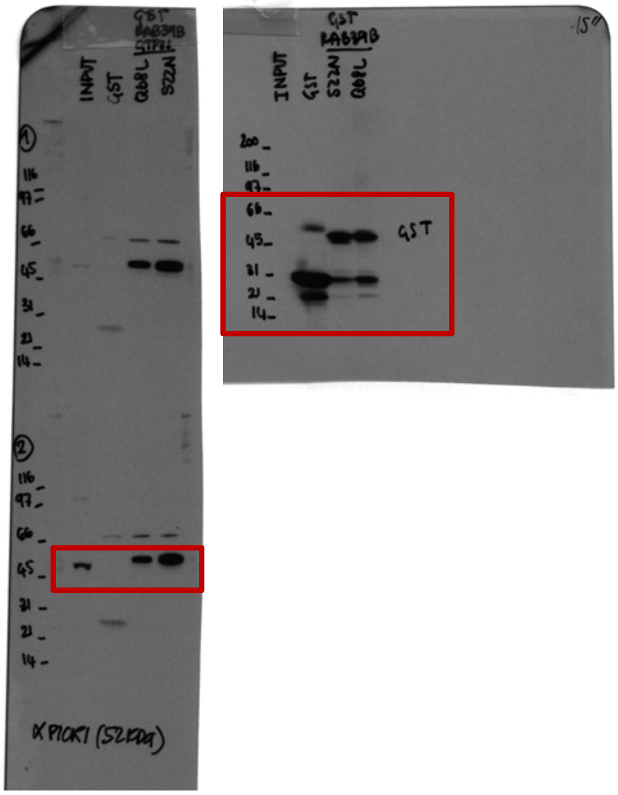

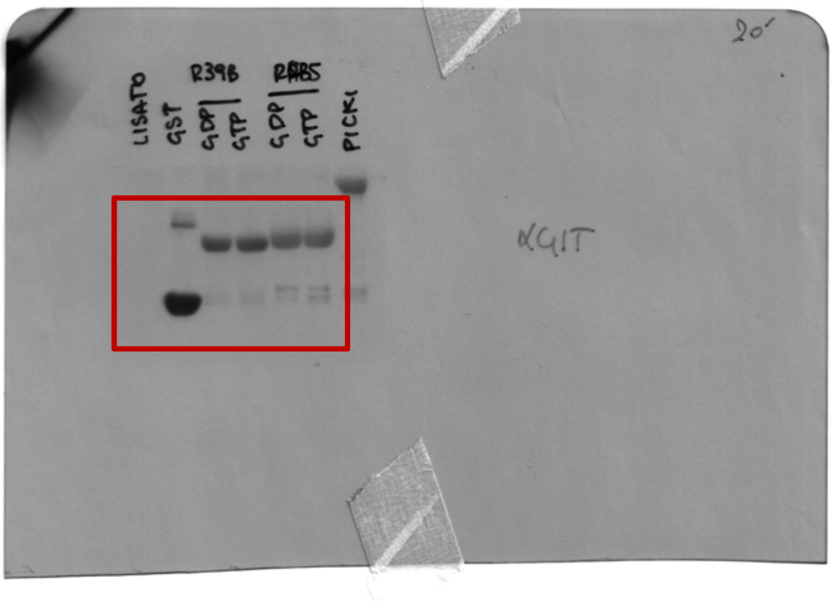

Supplementary Figure 6. Full scans of western blots from Figure 1a and 1b are shown

Fig.1c

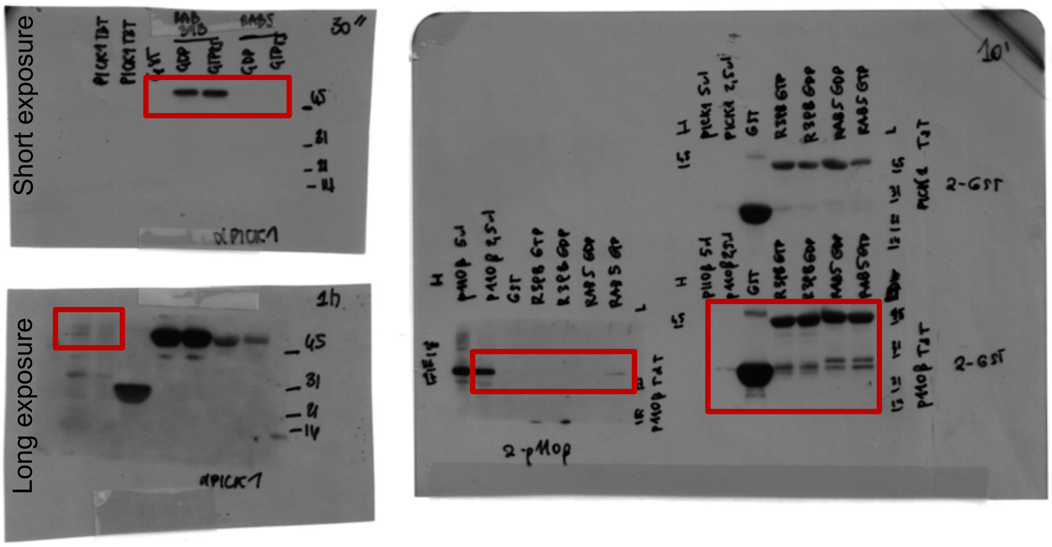

Fig.1d

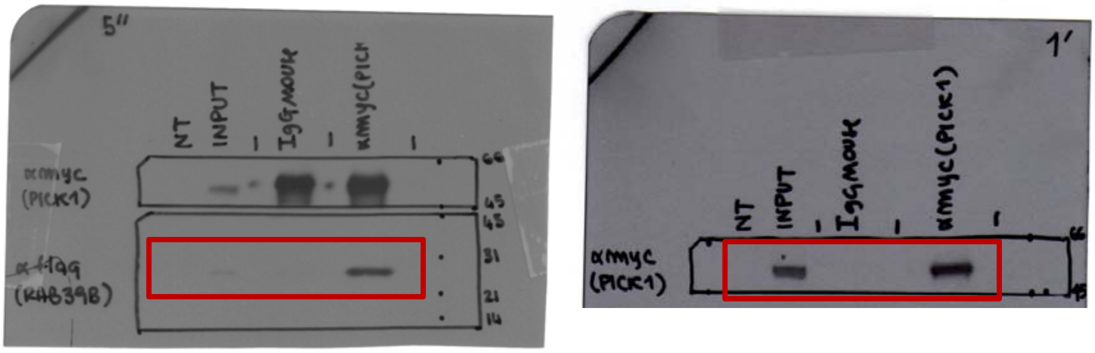

Supplementary Figure 6. Full scans of western blots from Figure 1c and 1d are shown

Fig.1e

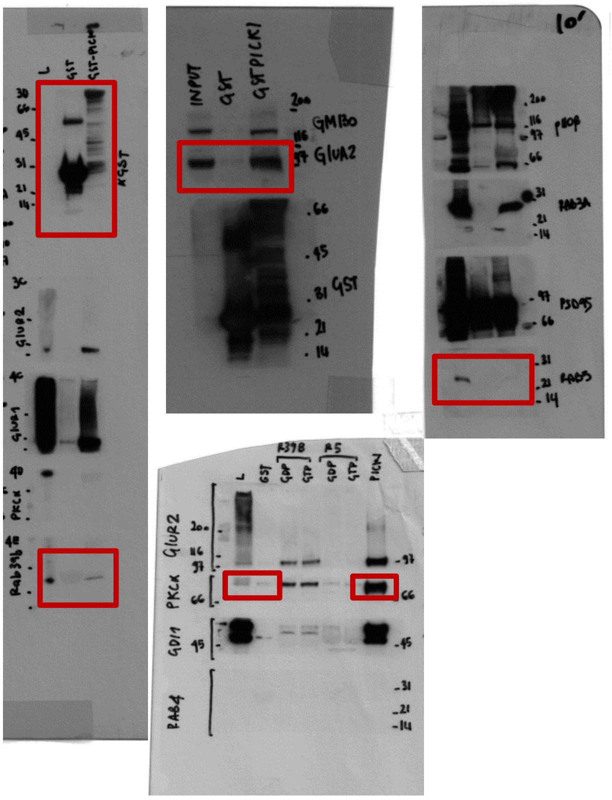

Supplementary Figure 6. Full scans of western blots from Figure 1e are shown

Fig.2a

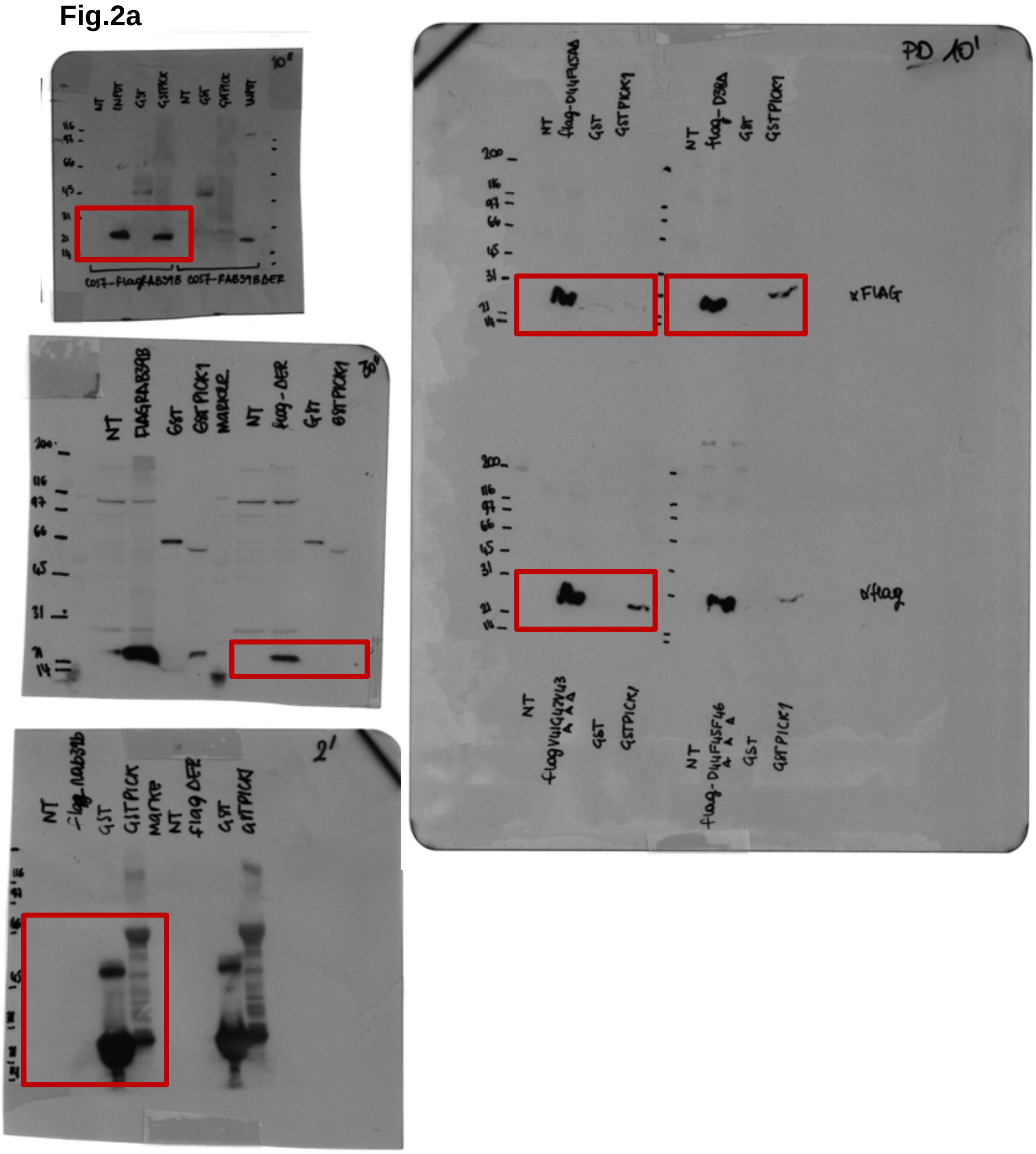

Supplementary Figure 6. Full scans of western blots from Figure 2a are shown

Fig.2b

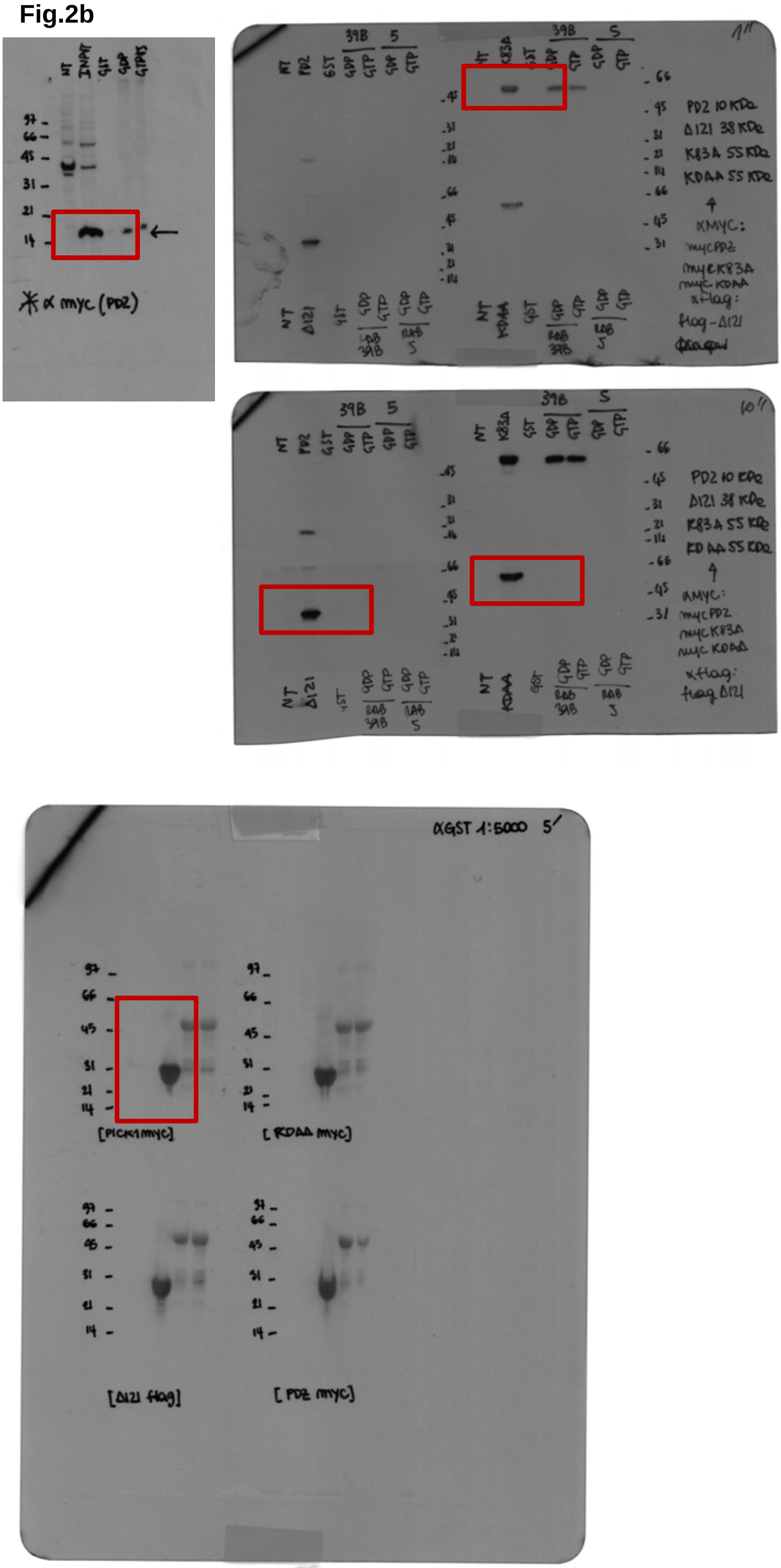

Supplementary Figure 6. Full scans of western blots from Figure 2b are shown

Fig. 5e

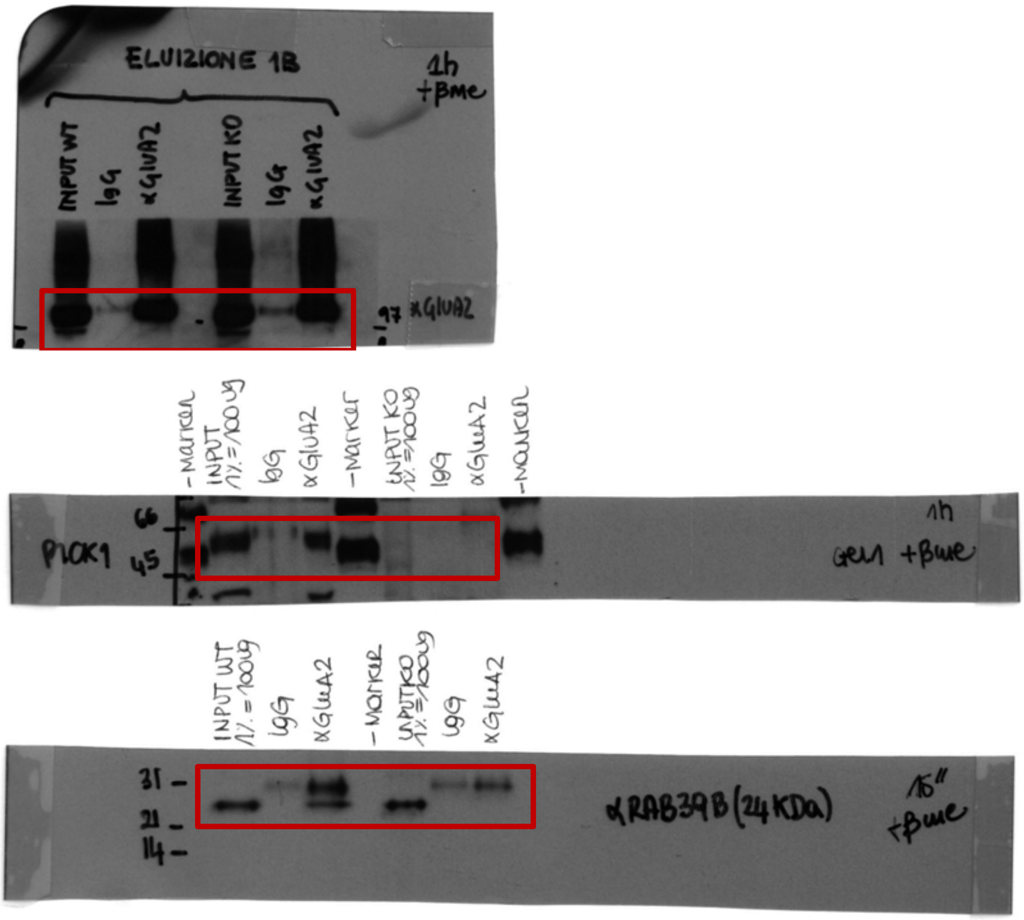

Supplementary Figure 6. Full scans of western blots from Figure 5e are shown

Fig.7b

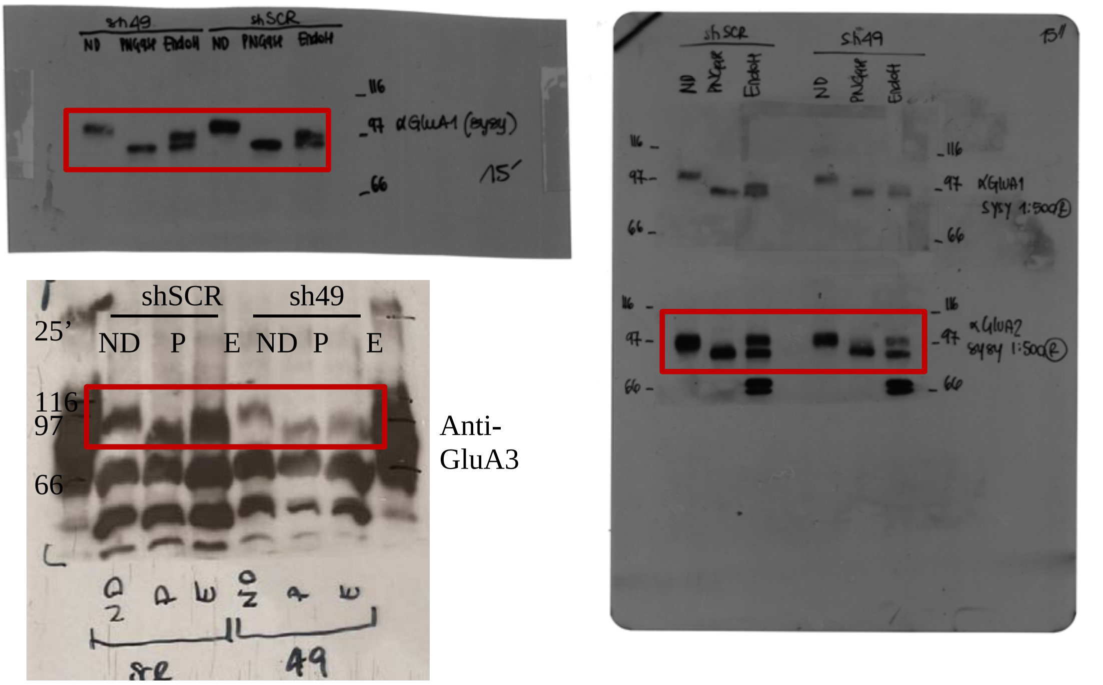

Supplementary Figure 6. Full scans of western blots from Figure 7b are shown

Supplementary Methods

E18 hippocampal neuronal cultures

Primary neuronal cultures were prepared from the hippocampi of E18 embryos from C57BL/6N mice as previously described [1]. After 15 minutes of incubation with 0.25% trypsin (Sigma) in Hanks' balanced salt solution (HBSS; Gibco) at 37°C, hippocampi were washed three times with HBSS to remove trypsin and then mechanically dissociated. Neurons stained with vital dye (Trypan Blue; Sigma-Aldrich) were counted and 150,000 cells/coverslip were plated on poly-L-lysine (Sigma-Aldrich; 0.1 mg/ml)-treated glass coverslips (15 mm diameter). Cells were plated in plating medium [modified Eagle's medium (MEM) supplemented with 10% horse serum (Gibco), 3.3 mM glucose and 2 mM glutamine (Lonza)] and incubated for 4 hours at 37°C in a 5% CO_2 humidified atmosphere to allow adhesion to the substrate. 4 hours after plating, coverslips were transferred into a cell-culture dish covered with a glia monolayer (prepared as described below) containing hippocampal medium [MEM supplemented with 1% N2 supplement (Invitrogen), 2 mM glutamine (Lonza), 1 mM sodium pyruvate and 4 mM glucose (Sigma-Aldrich)] conditioned for at least 6 hours. Coverslips were turned upside down, with neurons facing down, toward the astroglia.

Astroglial cell cultures

Astroglial cultures were prepared from brains of P2 (postnatal day 2) C57BL/6N mice as previously described [2]. The tissue was chopped into small pieces, washed with HBSS, then trypsinised in a solution containing 2.5% trypsin (Sigma-Aldrich) and 1 mg/ml DNAse (Sigma-Aldrich) for 15 minutes at 37°C. After the incubation cells were washed three times with HBSS and then dissociated. The cell suspension was let stand for 2 minutes in order to settle undissociated piece of tissues by gravity. Then the cells contained in the supernatant were plated in glial medium [MEM containing 10% horse serum, 0.6% glucose (Sigma-Aldrich), 2 mM glutamine, 100 IU/ml penicillin and 100 mg/ml streptomycin (Lonza)] and maintained at 37°C in a 5% CO_2 humidified atmosphere.

Electrophysiology in cultured hippocampal neurons

17-20 DIV hippocampal neurons transduced with shScramble or shRab39b or Rab39b rescue were recorded using the whole-cell patch-clamp configuration through a Multiclamp 700B amplifier (Axon instruments). Data were sampled at 10 kHz and digitized through a Digidata 1440A (Axon Instruments). Low resistance patch pipettes (3–5 MΩ), were filled with the following solution (126 mM K-gluconate, 4 mM NaCl, 10 mM HEPES, 10 mM glucose, 1 mM MgSO4, 0.5 mM CaCl21 EGTA, 3 mM MgATP, and 0.1 mM NaGTP, adjusted to pH 7.2 with KOH. The extracellular solution contained (140 mM NaCl, 3

mM KCl, 10 mM HEPES, 10 mM glucose, 2 mM $CaCl_2$, and 1.2 mM $MgCl_2$, adjusted to pH 7.4 with NaOH, maintained at 32°C). For recording miniature EPSCs (mEPSCs), cells were held at −70 mV in extracellular solution containing 3 μM TTX. mEPSCs were collected until the cell was stable using Clampex10 (Molecular Devices) and were analyzed using Clampfit10 (Molecular Devices). Threshold mEPSC amplitude was set at 5 pA, and 300–500 events were collected and averaged to calculate the mean mEPSC amplitude, frequency and kinetics for each culture preparation examined.

Electrophysiology in organotypic hippocampal slices

Hippocampal slice cultures were prepared from postnatal day 5-7 mice C57BL6. After 1 day in culture every slice was transduced with lentiviral particles expressing GFP plus shScramble or GFP plus shRab39b. After 10-12 days in culture, simultaneous voltage-clamp whole-cell recordings were obtained from nearby infected and uninfected CA1 pyramidal neurons under visual guidance using fluorescence and transmitted light illumination. Bipolar stimulating electrodes were placed over Schaffer collateral fibres between 250 and 500 μm from the CA1 recorded cells, and synaptic responses were evoked with single voltage pulses (100-300 μs, up to 20 V). The recording chamber was perfused with ACSF: 119 mM NaCl, 2.5 mM KCl, 4 mM $CaCl_2$, 4 mM $MgCl_2$, 26 mM $NaHCO_3$, 1 mM NaH_2PO_4, 11 mM glucose, 0.1 mM picrotoxin and 4 μM 2-chloroadenosine, pH 7.4, gassed with 5% CO_2/95% O_2. Patch recording pipettes (3–6 MΩ) were filled with 115 mM cesium methanesulfonate, 20 mM CsCl, 10 mM HEPES, 2.5 mM $MgCl_2$, 4 mM disodium ATP, 0.4 mM trisodium GTP, 10 mM sodium phosphocreatine and 0.6 mM EGTA, pH 7.25. For the rectification studies, AMPAR responses were recorded at −60 mV and +40 mV in the presence of 0.1 mM AP5 in the external solution and 0.1 mM spermine in the internal solution. The rectification index was calculated as the ratio between the AMPAR synaptic response at −60 mV and +40 mV. Synaptic responses were averaged over 50–100 trials. LTD induction was carried out by 500 stimuli at 1 Hz, with postsynaptic depolarization at -40 mV. Whole-cell recordings were made with a Multiclamp 700B amplifier (Axon Instruments) and electrophysiological data were collected with pCLAMP software (Molecular Devices).

Modeling a supramolecular complex, in which dimeric PICK1 acts as a scaffold for both RAB39B and GluA2-containingAMPAR.

Comparative modeling of PICK1

The structural model of PICK1 (i.e. sequence 14-347) was achieved by comparative modeling by means of the MODELLER software [3]. Comparative modeling relied on two templates, the crystal structure of the PICK1 PDZ domain fused with the C-terminal peptide of GluA2 (PDB code: 3HPK, chain A [4]) and the crystal structure of dimeric arfaptin-2 (PDB code: 4DCN, chain C [5]), which was employed to model

the BAR domain. The rationale for choosing the 3HPK structure is that it is the most complete in terms of resolved PDZ amino acids and the portion that precedes the GluA2 sequence (i.e. sequence 108-116) holds an α-helical structure. In that α-helical portion, the PDZ sequence ends to Q110, whereas the remaining amino acids belong to the peptide tethering PDZ to the C-term of GluA2. To model the link between the PDZ domain and the BAR domain, the GluA2 peptide was deleted and the terminal α-helical segment in the 3HPK template was prolonged by six turns holding the PICK1 sequence (i.e. until amino acid 139). It is worth noting that a combination of structure determinations [4] and secondary structure predictions (i.e. by a number of predictors including: Jpred, PSIPRED, SAM-T08, Raptor-X, and Phyre) suggest that the portion 108-138 may assume an α-helical conformation with most probable regions being 111-127 and 132-137. Thirteen different templates were probed, differing in the inclination and length of the α-helical link (i.e. the latter was also progressively deleted). For each template, 200 PICK1 models were generated by randomizing all the Cartesian coordinates of standard residues in the initial model, leading to a total of 2600 models, differing essentially in: a) the orientation of the PDZ domain with respect to the BAR domain, b) the coil-coil interactions between the N-terminal extension of helix 1 and the other two helices of the BAR domain, and c) in the conformation of the helix1/helix2 and helix2/helix3 loops of the BAR domain. The models were evaluated primarily according to the extent of violation of stereochemical restraints and according to the 3D-Profile score (i.e. computed within the Quanta package (www.accelrys.com)) that quantifies the goodness of sequence-to-fold fit. Successive selections were based on the evaluation of the stereochemical quality of backbone conformation. One chimeric template provided the most reliable models. Thirteen of these models were dimerized following fitting onto chains C and D of the arfaptin-2 dimer. [2]Using the amino acid side chain rotamer libraries implemented in the Quanta package relieved the few bad contacts at the inter-protomer faces.

Comparative modeling of RAB39B

The structural model of active RAB39B (sequence 7-178) was achieved by comparative modeling as well by using the crystal structure of active (i.e. GppNHp-bound) RAB8a in complex with the RAB binding domain of the 5-inositol phosphatase OCRL1 (PDB code: 3QTB, chain A [6]) as a template. The only two subtle structural differences between template and target proteins were predicted to fall in the β2/β3 turn and in the α3/β5 loop. In deep detail, as for the β2/β3 turn, whereas the inter-switch of RAB8a is a 2:2 β-hairpin, that of RAB39B is predicted to be a 3:5 β-hairpin. As for the α3/β5 loop, one-amino acid insertion is predicted in the RAB39B sequence. Thus, we probed nine different RAB8a templates holding various deletions in the turn and/or the loop. For each template and the corresponding template-target sequence alignment 100 models were built. The template structure

holding only a three-amino acid deletion (i.e. sequence 111-112) in the α3/β5 loop produced the most reliable structural models according to quality checks. The best one of these models was finally subjected to refinement of the β-hairpin portion comprising the three-amino acid turn and positions n-1 and c+1. The addition of β-sheet restraints, while refining, served to favor the optimization of the 3:5 type β-hairpin.

Assembling PICK1, RAB39B and homo-tetrameric GluA2 in a supramolecular complex

The complex between RAB39B and the PDZ domain of PICK1 was predicted by rigid-body protein-protein docking (i.e. by ZDOCK [7]). Since our in vitro experiments indicate that RAB39B recognizes the PDZ domain and not the BAR domain of PICK1, the isolated PDZ domain was used as a fixed protein (i.e. the target) whereas RAB39B was allowed to explore the roto-translational space around the PDZ surface (i.e. it acted as a probe). To improve sampling while reducing false positives, the 18-23, 57-79 and 96-104 portions of PDZ and the 78-150 portions of RAB39B were not considered, as their involvement in the interface would necessarily exclude those regions indicated as important for RAB39B-PDZ interaction. Remarkably, docking simulations without excluding those regions gave overlapping results with restricted sampling in terms of best and most populated docking modes, thus supporting the non-relevance of such regions for RAB39B recognition. To strengthen predictions, overcoming, at least in part, limitations due to the rigid-body approximation, the whole ensemble of 20 different PDZ structures from NMR determinations (i.e. PDB code 2PKU [8]) were probed. Each docking run was done in a "dense" sampling mode (i.e. a rotational sampling interval of 6º). The best 500 solutions from each of the 20 docking runs (i.e. according to the ZDOCK score) were merged together leading to a final pool of 10000 docking poses. Such predicted complexes were subjected to cluster analysis by means of the Wordom software [9] by using a Cα-atom RMSD cutoff of 7.5 Å. Remarkably, the best 10 hits from each run tend to fall in the most populated cluster that holds 68% of the 10000 solutions. The solution finally selected for the supramolecular assembly is the sixth best solution from the docking on the NMR model #17. Taken from granted that the RAB39B-PDZ interfaces in the complexes of cluster 1 are quite similar, our final selection privileged an interface in which D28 of PDZ was sufficiently close to R70 in swII of Rab39B to make a salt bridge interaction.

To preserve such interface, the 14-102 sequences of PDZ in the A and B chains of dimeric PICK1 were replaced by the 18-102 portion of model #17 from the 2PKU structure. Thus, the selected pose of RAB39B was added to the PDZ domain in chain A, whereas the GluA2Ct peptide from the NMR model #17 was added to the PDZ domain in chain B. The GTP molecule was added as well in RAB39B.

This operation was carried out for all the thirteen selected models of PICK1. Such complexes were subjected to side chain minimization by means of the CHARMM force field using an implicit water model [10]. Selection of the final model followed manual docking on the surface of a (1,2-dioleoyl-sn-glycero-3-phosphocholine (DOPC) liposome model with a 40 nm-diameter (Fig. 4A and B). This operation served to estimate the accomplishment of the proper membrane topology of the lipid binding motifs on both PDZ domains. Such a check clearly highlighted one model as the best.

To draw a more complete though rough picture of the supramolecular assembly the selected RAB39B:2PICK1:GluA2Ct was completed at the C-term of RAB39B and by linking the GluA2Ct to the transmembrane domain (TMD) of the GluA2 chain A from the crystal structure of the GluA2 homotetramer. The two C-terminal cysteines of RAB39B, i.e. C211 and C213 were S-geranylgeranylated.

In spite of the indeterminations of the non structured portions of the RAB39B C-term and of the CTD of GluA2, such a completion of the RAB39B:2PICK1:GluA2Ct complex served to provide further validation to the PICK1 model and its interaction mode with RAB39B, as well as to achieve a structure of framework for interpreting and designing novel *in vitro* experiments.

Supplementary References

1 Banker, G. A. & Cowan, W. M. Rat hippocampal neurons in dispersed cell culture. *Brain research* **126**, 397-342 (1977).

2 Kaech, S. & Banker, G. Culturing hippocampal neurons. *Nat Protoc* **1**, 2406-2415, doi:10.1038/nprot.2006.356 (2006).

3 Sali, A. & Blundell, T. L. Comparative protein modelling by satisfaction of spatial restraints. *J Mol Biol* **234**, 779-815, doi:10.1006/jmbi.1993.1626 (1993).

4 Shi, Y. *et al.* Redox-regulated lipid membrane binding of the PICK1 PDZ domain. *Biochemistry* **49**, 4432-4439, doi:10.1021/bi100269t (2010).

5 Nakamura, K. *et al.* Structural basis for membrane binding specificity of the Bin/Amphiphysin/Rvs (BAR) domain of Arfaptin-2 determined by Arl1 GTPase. *The Journal of biological chemistry* **287**, 25478-25489, doi:10.1074/jbc.M112.365783 (2012).

6 Hou, X. *et al.* A structural basis for Lowe syndrome caused by mutations in the Rab-binding domain of OCRL1. *The EMBO journal* **30**, 1659-1670, doi:10.1038/emboj.2011.60 (2011).

7 Mintseris, J. *et al.* Integrating statistical pair potentials into protein complex prediction. *Proteins* **69**, 511-520, doi:10.1002/prot.21502 (2007).

8 Pan, L. *et al.* Clustering and synaptic targeting of PICK1 requires direct interaction between the PDZ domain and lipid membranes. *The EMBO journal* **26**, 4576-4587, doi:10.1038/sj.emboj.7601860 (2007).

9 Seeber, M. *et al.* Wordom: a user-friendly program for the analysis of molecular structures, trajectories, and free energy surfaces. *J Comput Chem* **32**, 1183-1194, doi:10.1002/jcc.21688 (2011).

10 Im, W., Lee, M. S. & Brooks, C. L., 3rd. Generalized born model with a simple smoothing function. *J. Comput. Chem.* **24**, 1691-1702 (2003).
